# *Xylaria furcata* reconsidered and nine resembling species

**DOI:** 10.1186/s40529-023-00392-x

**Published:** 2023-07-17

**Authors:** Yu-Ming Ju, Huei-Mei Hsieh, Nuttika Suwannasai

**Affiliations:** 1 Institute of Plant and Microbial Biology, Academia Sinica, Nankang, Taipei, 11529 Taiwan; 2 Department of Microbiology, Faculty of Science, Srinakharinwirot University, 114 Sukhumvit 23, Wathana, Bangkok, 10110 Thailand

**Keywords:** Systematics, Termite nests, Xylariaceae

## Abstract

**Background:**

*Xylaria* collections from termite nests with dichotomously branched stromata have been identified as *X*. *furcata*. However, Léveillé’s original material is no longer available, and the modern interpretation of *X*. *furcata* is based on a 1908 collection made by von Höhnel from termite nests at Buitenzorg Botanical Garden in Java. A packet of this von Höhnel material at FH was designated as the neotype by Rogers et al. in 2005.

**Results:**

We reexamined the neotype from FH and its duplicates from various herbaria and found that three different species were mixed in these specimens. Despite that all of them have dichotomously branched stromata and tiny ascospores, only one fits the 2005 neotypification of *X*. *furcata*, where exposed perithecial mounds on the stromatal surface were unambiguously indicated. This portion of material is redesignated as the neotype, while the other two species with immersed perithecia are described as new: *X*. *hoehnelii* and *X*. *robustifurcata*. The ITS sequence obtained from the neotype helped us designate a specimen with cultures obtained from it as the epitype. From specimens identifiable as *X*. *furcata*, we describe four new species: *X*. *brevifurcata*, *X*. *furcatula*, *X*. *insignifurcata*, and *X*. *tenellifurcata*. Additionally, we recognize *X*. *furcata* var. *hirsuta* at the species level as *X*. *hirsuta* and consider *X*. *scoparia* a distinct species rather than a synonym of *X*. *furcata*. Molecular phylogenetic analyses based on three protein-coding loci showed that *X*. *furcata* and resembling species were grouped into two clusters: the *X*. *furcata* cluster with half-exposed to fully exposed perithecial mounds and the *X*. *hoehnelii* cluster with largely immersed perithecial mounds.

**Conclusion:**

Ten species are recognized for *X. furcata* and resembling species, all of which could have been identified as *X*. *furcata* in the past. Its diversity has been overlooked primarily due to the small and similar stromata. Several additional species have been confirmed to be related to *X*. *furcata* by DNA sequences but are yet to be described due to the lack of mature stromata. While the species diversity of macrotermitine termites is equally high in Africa as in Asia, all of the species are primarily found in Asia, with *X*. *hirsuta* as the only exception. This suggests that there may be many more undiscovered species for this fungal group.

**Supplementary Information:**

The online version contains supplementary material available at 10.1186/s40529-023-00392-x.

## Background

Specimens of *Xylaria* Hill ex Schrank collected from termite nests and featuring antler-like dichotomously branched stromata have commonly been referred to as *X. furcata* Fr. This name was proposed by Fries (1851) to replace *Sphaeria dichotoma* Lév., which was published by Léveillé (1845) on the basis of a collection made by Pieter Willem Korthals from Java, Indonesia. Fries (1851) replaced *S*. *dichotoma* with *X*. *furcata* because he had recombined the epithet of *Hypoxylon dichotomum* Mont. with *Xylaria* to form *X*. *dichotoma* (Mont.) Fr. and thus could not recombine *S*. *dichotoma* with *Xylaria*, which would have created a later homonym. Léveillé (1845) compared *S*. *dichotoma* with *S*. *scopiformis* Kunze, an invalidly published name based on a Suriname collection made by Christoph Weigelt in 1827, and stated that both are sterile. The herbarium of Joseph-Henri Léveillé was destroyed during the Franco-Prussian war in 1870–1871. Searching for the Léveillé material of *S*. *dichotoma* at L and PC or the Fries material of *X. furcata* at UPS was unfortunately fruitless (Rogers et al. 2005).

The modern interpretation of *X*. *furcata* is rooted in von Höhnel (1908) who believed that he had collected *X*. *furcata* from termite nests at Buitenzorg Botanical Garden of Java, currently Bogor Botanical Gardens, regardless of the rather vague account on *X. furcata* [≡ *S*. *dichotoma*] in Léveillé (1845), where the Korthals material was recorded as being immature and associated with trunk. von Höhnel (1908) sent portions of his ample *X*. *furcata* collection made during 1907–1908 from Buitenzorg Botanical Garden to his contemporaries Heinrich Rehm and Giacomo Bresadola and stated that his interpretation of *X*. *furcata* was agreed by both of them. Rehm kept a packet for his own herbarium and distributed the rest of the von Höhnel collection as “1812. *Xylaria* (*Xylostyla*) *furcata* Fr.” in the exsiccati *Ascomyceten* that he issued. A packet of this von Höhnel material at FH was designated as the neotype by Rogers et al. (2005).

Petch (1906), who studied *X. furcata* in Sri Lanka, believed that there was only one *Xylaria* species from termite nests, *X. nigripes* (Klotsch) M. C. Cooke, and thought that it could have many morphs, with stromata branched or unbranched, robust or delicate, acute or rounded at apices. However, Petch (1913) changed his earlier view of one-species concept and accepted *X. furcata* as a separate species from *X*. *nigripes*, apparently influenced by von Höhnel (1908). Nonetheless, the two-species concept of von Höhnel (1908) still underestimated the diversity of *Xylaria* species from termite nests. An example can be found in his collection of *X*. *furcata* from Buitenzorg Botanical Garden, which contains stromata quite variable in shape and provide a clue to the mixed nature of his collection, as shown in Plates 3 and 4, in von Höhnel (1908). After studying the parts of the von Höhnel collection of *X*. *furcata* stored in FH, K, and S, we identified four different species: *X*. *furcata*, *X*. *scoparia* Pat., and two species described as new herein: *X*. *hoehnelii* and *X*. *robustifurcata*.

While studying Xylariaceae in Congo, Dennis (1961) stated that he followed Petch’s concept of *X*. *furcata*, but the cited specimen *Louis 14866* is unlike Petch’s material from Sri Lanka in having hirsute stromata. Like Petch’s material, von Höhnel’s *X*. *furcata* from Indonesia also has glabrous stromata. Therefore, Rogers et al. (2005) described *Louis 14866* as *X*. *furcata* var. *hirsuta* J. D. Rogers & Y.-M. Ju.

We studied numerous specimens that were identified or suspected as *X*. *furcata* and obtained cultures from viable ones. After examining their teleomorphic and anamorphic characteristics and analyzing the sequences of three protein-coding loci and ITS, we concluded that *Xylaria* species from termite nests with delicate forked stromata consist of ten different species. Among these, only *X*. *furcata*, its var. *hirsuta*, and *X*. *scoparia* haven been described, while others are newly described herein. The neotype designated for *X*. *furcata* in Rogers et al. (2005) should be reconsidered because it was based on a Höhnel packet in FH containing more than one species.

## Methods

### Fungal materials and observations

Specimens labeled as *X*. *furcata* were loaned from various herbaria. Culturable materials identifiable as *X*. *furcata* were collected from nesting sites of *Odontotermes formosanus* in Taiwan. Some of the stromata are fully mature, bearing asci and ascospores, while others are sterile or bear conidia only. Fresh stromatal tissue was placed on SME medium (Kenerley and Rogers 1976) to obtain cultures. If cultures could not be isolated in a timely manner, freshly collected stromata were stored in a moist condition in the fridge to prevent drying and mold growth. Resulting colonies were transferred to 9-cm plastic Petri dishes containing 2% Difco oatmeal agar (OMA) for culture descriptions and incubated at 20 °C under 12 h fluorescent light. Asci, ascospores, conidiogenous cells, and conidia were examined using differential interference contrast microscopy (DIC) and bright field microscopy (BF). Material was mounted in water and Melzer’s iodine reagent for examination by DIC and BF. Cultures were deposited at BCRC (the Bioresource Collection and Research Center, Hsin-chu, Taiwan). Only a culture was deposited if cultures were obtained from multiple stromata of a specimen. Those stromata with cultures isolated or DNAs extracted were each labeled with a number prefixed with “YMJ.”

### Phylogenetic analyses

Sequences of the loci for β-tubulin (*β-TUB*) and α-actin (*α-ACT*) were obtained from *X*. *furcata* and resembling species following Hsieh et al. (2005), while those of the loci for the second largest subunit of RNA polymerase II (*RPB2*) and nuc rDNA internal transcribed spacers (ITS = ITS1-5.8S-ITS2) were obtained following Hsieh et al. (2010) and Hsieh et al. (2009), respectively. Obtained sequences from *X*. *furcata* and resembling species were listed in Table 1 and deposited at GenBank. Other sequences included in the phylogenetic analyses were listed in Additional file 1: Table S1.

**Table 1:** Species and isolates of the *X*. *furcata* and resembling species included in the phylogenetic analyses. Sequences in boldface were generated in this study

Taxon	Origin	Collecting data	GenBank Accession number			
			β-tubulin gene	α-actin gene	RPB2 gene	ITS
*X. brevifurcata* Y.-M. Ju & H.-M. Hsieh	Taiwan	*YMJ986*	**OQ818431**	**OQ818371**	**OQ851525**	**OQ845481**
*X. brevifurcata* Y.-M. Ju & H.-M. Hsieh	Taiwan	*YMJ1381* from HOLOTYPE	**OQ818432**	**OQ818372**	**OQ851526**	**OQ845482**
*X. furcata* Fr.	Indonesia	From NEOTYPE	–	–	–	**OQ848122**
*X. furcata* Fr.	Indonesia	Stromata emerging from fungus comb (FH)	–	–	–	**OQ848123**
*X. furcata* Fr.	Taiwan	*YMJ646*, as *X*. sp. 4 in Hsieh et al. (2010)	GQ502715	GQ853050	GQ853032	GU324757
*X. furcata* Fr.	Taiwan	*YMJ956*	**OQ818449**	**OQ818389**	**OQ851543**	**OQ842973**
*X. furcata* Fr.	Taiwan	*YMJ978*	**OQ818450**	**OQ818390**	**OQ851544**	**OQ842974**
*X. furcata* Fr.	Taiwan	*YMJ979*	**OQ818451**	**OQ818391**	**OQ851545**	**OQ842975**
*X. furcata* Fr.	Taiwan	*YMJ990*	**OQ818452**	**OQ818392**	**OQ851546**	**OQ842976**
*X. furcata* Fr.	Taiwan	*YMJ993*	**OQ818453**	**OQ818393**	**OQ851547**	**OQ842977**
*X. furcata* Fr.	Taiwan	*YMJ994*	**OQ818454**	**OQ818394**	**OQ851548**	**OQ842978**
*X. furcata* Fr.	Taiwan	*YMJ1100*	**OQ818455**	**OQ818395**	**OQ851549**	**OQ842979**
*X. furcata* Fr.	Taiwan	*YMJ1101*	**OQ818456**	**OQ818396**	**OQ851550**	**OQ842980**
*X. furcata* Fr.	Taiwan	*YMJ1102*	**OQ818457**	**OQ818397**	**OQ851551**	**OQ842981**
*X. furcata* Fr.	Taiwan	*YMJ1120*	**OQ818458**	**OQ818398**	**OQ851552**	**OQ842982**
*X. furcata* Fr.	Taiwan	*YMJ1121*	**OQ818459**	**OQ818399**	**OQ851553**	**OQ842983**
*X. furcata* Fr.	Taiwan	*YMJ1122*	**OQ818460**	**OQ818400**	**OQ851554**	**OQ842984**
*X. furcata* Fr.	Taiwan	*YMJ1123*	**OQ818461**	**OQ818401**	**OQ851555**	**OQ842985**
*X. furcata* Fr.	Taiwan	*YMJ1125*	**OQ818462**	**OQ818402**	**OQ851556**	**OQ842986**
*X. furcata* Fr.	Taiwan	*YMJ1126*	**OQ818463**	**OQ818403**	**OQ851557**	**OQ842987**
*X. furcata* Fr.	Taiwan	*YMJ1127*	**OQ818464**	**OQ818404**	**OQ851558**	**OQ842988**
*X. furcata* Fr.	Taiwan	*YMJ1128*	**OQ818465**	**OQ818405**	**OQ851559**	**OQ842989**
*X. furcata* Fr.	Taiwan	*YMJ1129*	**OQ818466**	**OQ818406**	**OQ851560**	**OQ842990**
*X. furcata* Fr.	Taiwan	*YMJ1132*	**OQ818467**	**OQ818407**	**OQ851561**	**OQ842991**
*X. furcata* Fr.	Taiwan	*YMJ1133*	**OQ818468**	**OQ818408**	**OQ851562**	**OQ842992**
*X. furcata* Fr.	Taiwan	*YMJ1134*	**OQ818469**	**OQ818409**	**OQ851563**	**OQ842993**
*X. furcata* Fr.	Taiwan	*YMJ1135*	**OQ818470**	**OQ818410**	**OQ851564**	**OQ842994**
*X. furcata* Fr.	Taiwan	*YMJ1730* from EPITYPE	**OQ818471**	**OQ818411**	**OQ851565**	**OQ842995**
*X. furcata* Fr.	Taiwan	*YMJ1731* from EPITYPE	**OQ818472**	**OQ818412**	**OQ851566**	**OQ842996**
*X. furcata* Fr.	Taiwan	*YMJ1732* from EPITYPE	**OQ818473**	**OQ818413**	**OQ851567**	**OQ842997**
*X. furcata* Fr.	Taiwan	*YMJ1736* from EPITYPE	**OQ818474**	**OQ818414**	**OQ851568**	**OQ842998**
*X. furcata* Fr.	Taiwan	*YMJ1737* from EPITYPE	**OQ818475**	**OQ818415**	**OQ851569**	**OQ842999**
*X. furcata* Fr.	Taiwan	*YMJ1738* from EPITYPE	**OQ818476**	**OQ818416**	**OQ851570**	**OQ843000**
*X. furcatula* Y.-M. Ju & H.-M. Hsieh	Taiwan	*YMJ1099* from HOLOTYPE	**OQ818433**	**OQ818373**	**OQ851527**	**OQ845483**
*X. furcatula* Y.-M. Ju & H.-M. Hsieh	Taiwan	*YMJ1103* from HOLOTYPE	**OQ818434**	**OQ818374**	**OQ851528**	**OQ845484**
*X. furcatula* Y.-M. Ju & H.-M. Hsieh	Taiwan	*YMJ1916*	**OQ818435**	**OQ818375**	**OQ851529**	**OQ845485**
*X. furcatula* Y.-M. Ju & H.-M. Hsieh	Taiwan	*YMJ1917*	**OQ818436**	**OQ818376**	**OQ851530**	**OQ845486**
*X. furcatula* Y.-M. Ju & H.-M. Hsieh	Taiwan	*YMJ1918*	**OQ818437**	**OQ818377**	**OQ851531**	**OQ845487**
*X. furcatula* Y.-M. Ju & H.-M. Hsieh	Taiwan	*YMJ2194*	**OQ818438**	**OQ818378**	**OQ851532**	**OQ845488**
*X. hoehnelii* Y.-M. Ju & H.-M. Hsieh	Taiwan	*YMJ642* from HOLOTYPE, as *X*. sp. 1 in Hsieh et al. (2010)	GQ502719	GQ853054	GQ853036	GU324759
*X. hoehnelii* Y.-M. Ju & H.-M. Hsieh	Taiwan	*YMJ643* from HOLOTYPE	**OQ818439**	**OQ818379**	**OQ851533**	**OQ845489**
*X. hoehnelii* Y.-M. Ju & H.-M. Hsieh	Taiwan	*YMJ644* from HOLOTYPE	**OQ818440**	**OQ818380**	**OQ851534**	**OQ845490**
*X. hoehnelii* Y.-M. Ju & H.-M. Hsieh	Taiwan	*YMJ645* from HOLOTYPE	**OQ818441**	**OQ818381**	**OQ851535**	**OQ845491**
*X. hoehnelii* Y.-M. Ju & H.-M. Hsieh	Taiwan	*YMJ1151*	**OQ818442**	**OQ818382**	**OQ851536**	**OQ845492**
*X. hoehnelii* Y.-M. Ju & H.-M. Hsieh	Taiwan	*YMJ1159*	**OQ818443**	**OQ818383**	**OQ851537**	**OQ845493**
*X. hoehnelii* Y.-M. Ju & H.-M. Hsieh	Taiwan	*YMJ2178*	**OQ818444**	**OQ818384**	**OQ851538**	**OQ845494**
*X. hoehnelii* Y.-M. Ju & H.-M. Hsieh	Taiwan	*YMJ2179*	**OQ818445**	**OQ818385**	**OQ851539**	**OQ845495**
*X. insignifurcata* Y.-M. Ju & H.-M. Hsieh	Taiwan	*YMJ650* from HOLOTYPE, as *X*. sp. 5 in Hsieh et al. (2010)	GQ502716	GQ853051	GQ853033	GU324758
*X. insignifurcata* Y.-M. Ju & H.-M. Hsieh	Taiwan	*YMJ649* from HOLOTYPE	**OQ818446**	**OQ818386**	**OQ851540**	**OQ845496**
*X. insignifurcata* Y.-M. Ju & H.-M. Hsieh	Taiwan	*YMJ1198*	**OQ818447**	**OQ818387**	**OQ851541**	**OQ845497**
*X. scoparia* Pat.	Taiwan	*YMJ958*	**OQ818477**	**OQ818417**	**OQ851571**	**OQ843001**
*X. scoparia* Pat.	Taiwan	*YMJ959*	–	–	–	**OQ843002**
*X. scoparia* Pat.	Taiwan	*YMJ960*	**OQ818478**	**OQ818418**	**OQ851572**	**OQ843003**
*X. scoparia* Pat.	Taiwan	*YMJ982*	**OQ818479**	**OQ818419**	**OQ851573**	**OQ843004**
*X. scoparia* Pat.	Taiwan	*YMJ983*	**OQ818480**	**OQ818420**	**OQ851574**	**OQ843005**
*X. scoparia* Pat.	Taiwan	*YMJ989*	**OQ818481**	**OQ818421**	**OQ851575**	**OQ843006**
*X. scoparia* Pat.	Taiwan	*YMJ991*	**OQ818482**	**OQ818422**	**OQ851576**	**OQ843007**
*X. scoparia* Pat.	Taiwan	*YMJ992*	**OQ818483**	**OQ818423**	**OQ851577**	**OQ843008**
*X. scoparia* Pat.	Taiwan	*YMJ1158*	**OQ818484**	**OQ818424**	**OQ851578**	**OQ843009**
*X. scoparia* Pat.	Taiwan	*YMJ1423*	**OQ818485**	**OQ818425**	**OQ851579**	**OQ843010**
*X. scoparia* Pat.	Taiwan	*YMJ1424*	**OQ818486**	**OQ818426**	**OQ851580**	**OQ843011**
*X. scoparia* Pat.	Taiwan	*YMJ1427*	**OQ818487**	**OQ818427**	**OQ851581**	**OQ843012**
*X. scoparia* Pat.	Taiwan	*YMJ1428*	**OQ818488**	**OQ818428**	**OQ851582**	**OQ843013**
*X. scoparia* Pat.	Taiwan	*YMJ1435* from EPITYPE	**OQ818489**	**OQ818429**	**OQ851583**	**OQ843014**
*X. scoparia* Pat.	Taiwan	*YMJ2177*	**OQ818490**	**OQ818430**	**OQ851584**	**OQ843015**
*X. siamensis* Wangsawat et al.	Thailand	*SWUF17-20.2* from HOLOTYPE (Wangsawat et al. 2021)	**OQ845437**	**OQ845429**	**OQ851589**	MT622765
*X. tenellifurcata* Y.-M. Ju & H.-M. Hsieh	Taiwan	*YMJ1070* from HOLOTYPE	**OQ818448**	**OQ818388**	**OQ851542**	**OQ845498**

The ITS dataset (Additional file 2) was formed using ITS sequences from *X*. *furcata* and resembling species as well as the neotype of *X*. *furcata*, with *X*. *hypoxylon* (L.) Grev. as the outgroup. The concatenated dataset RPB2-TUB-ACT (Additional file 3) was formed using sequences of *α-ACT*, *RPB2*, and *β-TUB* from *X*. *furcata* and resembling species (Table 1) as well as various species of *Xylaria* and closely related genera of *Xylaria* (Additional file 1: Table S1), with *Biscogniauxia arima* F. San Martín, Y.-M. Ju & J. D. Rogers as the outgroup. Both datasets were aligned using Clustal X 1.81 (Thompson et al. 1997) with the “gap penalty” set to 10 and “gap extension penalty” set to 0.2, and improved manually.

To calculate the similarities between pairs of ITS sequences from *X. furcata* and resembling species, we used DNADIST from the PHYLIP version 3.6 phylogenetic inference package (Felsenstein 2005).

Maximum-Likelihood (ML) and Bayesian Inference (BI) analyses were performed on the aligned ITS dataset (Additional file 2) and the aligned RPB2-TUB-ACT dataset (Additional file 3). ML trees were generated using RAxML analysis ver. 8.2.10 (Stamatakis 2014) with rapid bootstrap support and 1000 replicates of bootstrap test to assess the robustness of the tree topology. BI trees were generated using MrBayes ver. 3.2.6 (Ronquist et al. 2012) with a Markov Chain Monte Carlo (MCMC) algorithm. Four MCMC chains (one cold and three heated) were run for one million generations with the trees sampled every 100 generations to ensure convergence of the chains. The first 25% trees were excluded as burn-in phases, and posterior probability values were estimated with the 75% remaining trees to assess the support for each clade in the tree. Models of evolution for both ML and BI trees were defined by MrModeltest 2.4 (Nylander 2004), and consensus trees were viewed in FigTree ver. 1.4.4 (http://tree.bio.ed.ac.uk/software/figtree/).

## Results

### Concept of *X. furcata* reconfirmed through ITS sequence analysis of the neotype and comparison of ITS similarities between species pairs

**Fig. 1:**
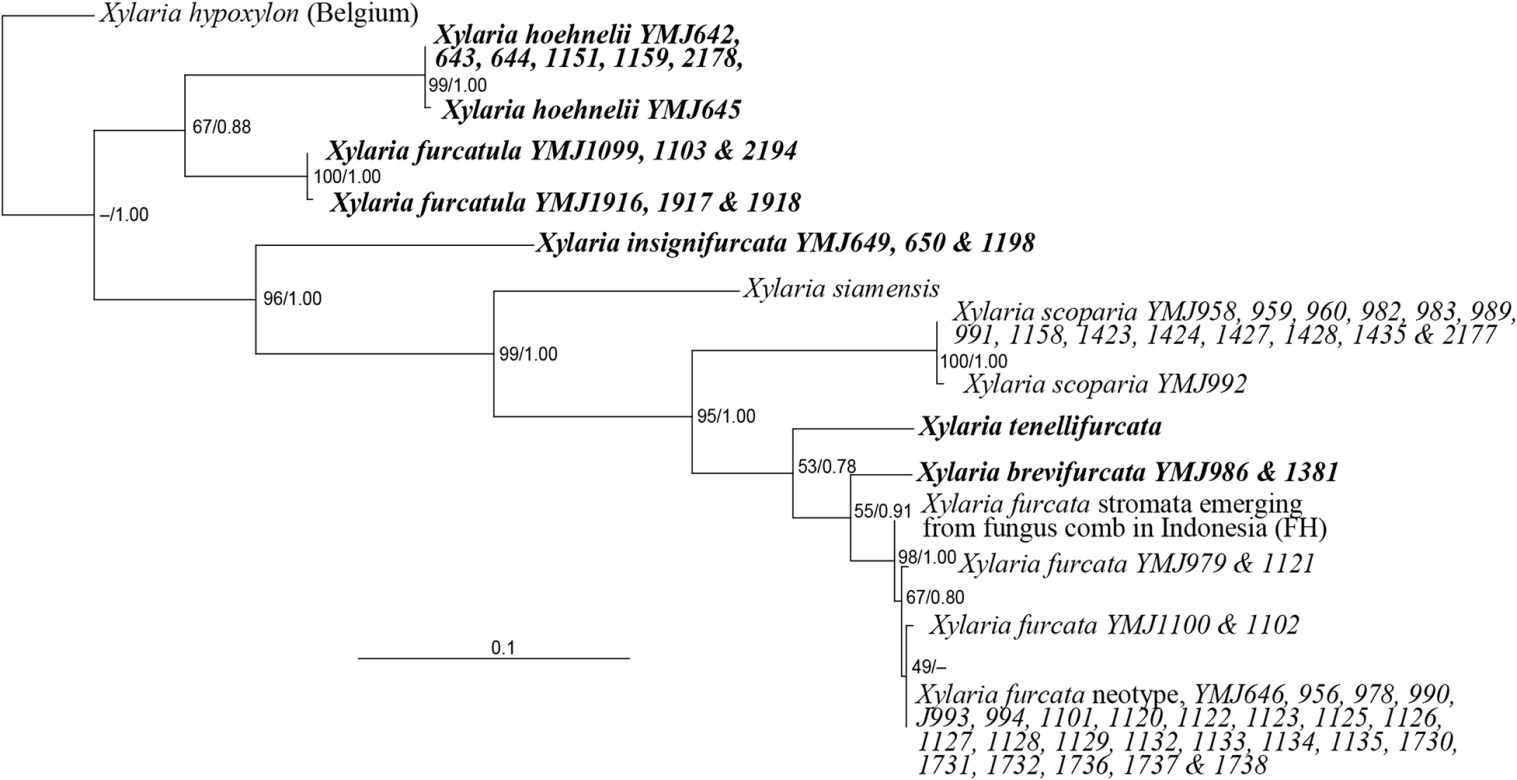
Phylogenetic tree generated by ML analysis from the ITS dataset with the sequence from the neotype of *X*. *furcata* included. The species newly described in the present study are in boldface. Numbers at internodes represent bootstrap values and are immediately followed by the posterior probability values greater than 50% with BI analysis. *Xylaria hypoxylon* is the outgroup taxon

The ITS sequences from the neotype of *X*. *furcata* and specimens identifiable as *X*. *furcata* (Table 1) were analyzed using BI and ML phylogenetic methods. Both analyses generated largely congruent trees, and only the ML tree is shown (Fig. 1). The ITS sequence from the neotype of *X*. *furcata* matched sequences from cultures obtained from stromata of nine specimens, including *YMJ646* of specimen *95072001*, *YMJ990, 993 & 994* of specimen *97071501*, *YMJ978* of specimen *97072705*, *YMJ1101* of specimen *98070101*, *YMJ1120, 1122 & 1123* of specimen *98072301*, *YMJ1125–1129* of specimen *98072401*, *YMJ956 & 1135* of specimen *98073103*, *YMJ1132–1134* of specimen *98080301*, and *YMJ1730–1732* and *1736–1738* of specimen *104072801*. However, slight differences (> 99.6% similarity) were observed with sequences from three of the nine stromata, including *YMJ979* of specimen *97072705*, *YMJ1100 & 1102* of specimen *98070101*, and *YMJ1121* of specimen *98072301*. Specimen *104072801* contains ample material and is designated as the epitype of *X*. *furcata* herein. A packet attached on the same herbarium sheet as the neotype from FH containing immature stromata emerging from a fungus comb was also confirmed to be *X. furcata*.

The ITS similarities between *X*. *furcata* and resembling species are shown in Table 2. The species most similar to *X*. *furcata* is *X*. *brevifurcata* with a similarity range of 96.5–97.0%, followed by *X*. *tenellifurcata* with a similarity range of 94.1–94.3%, and *X*. *scoparia* with a similarity range of 91.2–91.5%. The similarities between other pairs of species are less than 93.80%, which is the similarity shared between *X*. *brevifurcata* and *X*. *tenellifurcata*.

**Table 2:** Comparison of ITS similarities between species pairs of *X. furcata* and resembling species

Taxon	*X. brevifurcata* (%)	*X. furcata* (%)	*X. furcatula* (%)	*X. hoehnelii* (%)	*X. insignifurcata* (%)	*X. scoparia* (%)	*X. siamensis* (%)	*X. tenellifurcata* (%)
*X. brevifurcata*	100							
*X. furcata*	96.5–97.0	99.6–100						
*X. furcatula*	84.0–84.2	84.2–84.6	99.8–100					
*X. hoehnelii*	83.7	83.1–83.5	89.3–89.7	99.8–100				
*X. insignifurcata*	85.6	85.2–85.4	86.9–87.1	86.9–87.1	100			
*X. scoparia*	91.4	91.2–91.5	83.2–83.4	83.2	84.5	99.8–100		
*X. siamensis*	87.7	88.3–88.5	83.3–83.5	84.0–84.2	85.8	87.2	100	
*X. tenellifurcata*	93.8	94.1–94.3	83.5–83.8	84.2–84.4	85.2	90.3	89.1	100

### Molecular phylogenetic analyses based on the RPB2-TUB-ACT dataset

**Fig. 2:**
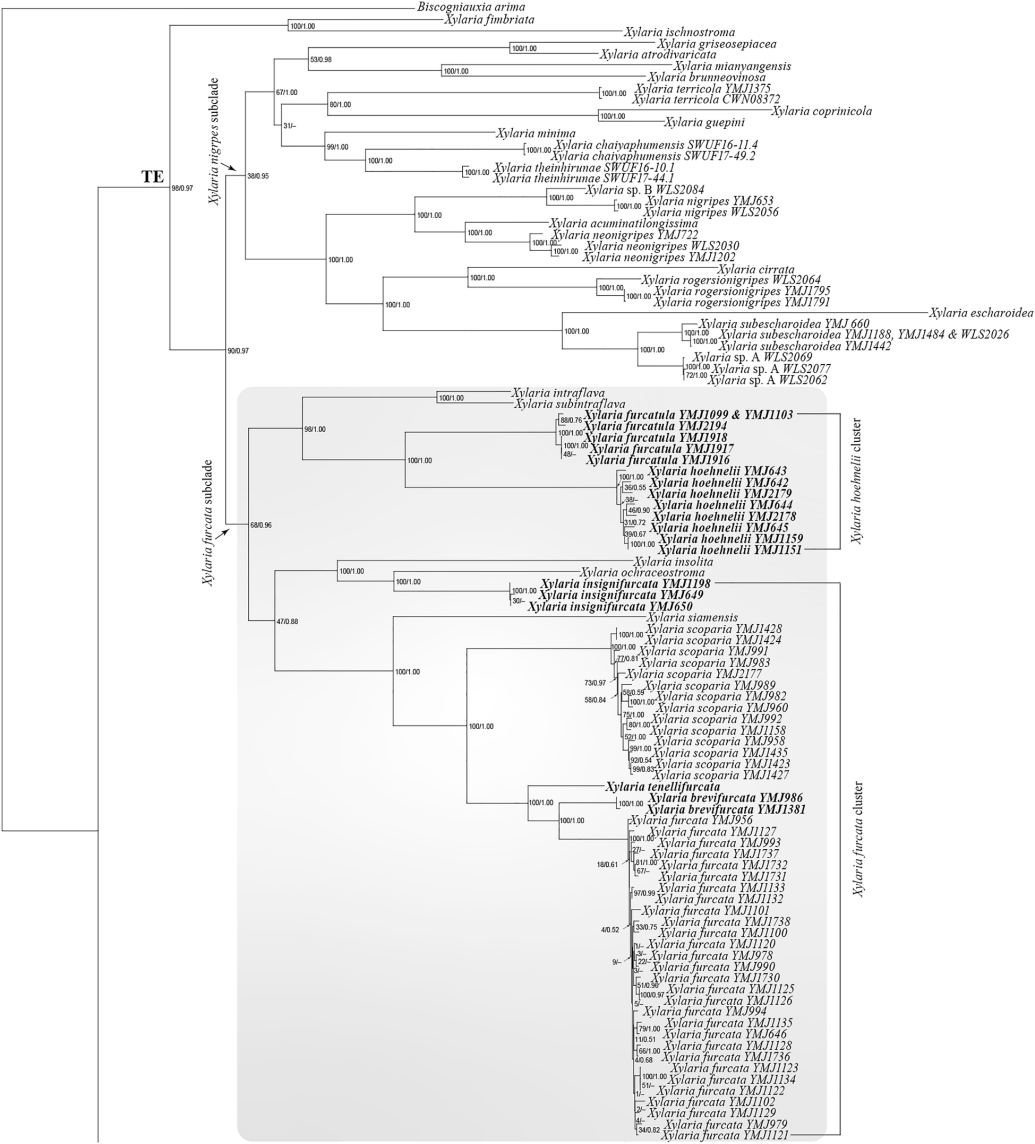

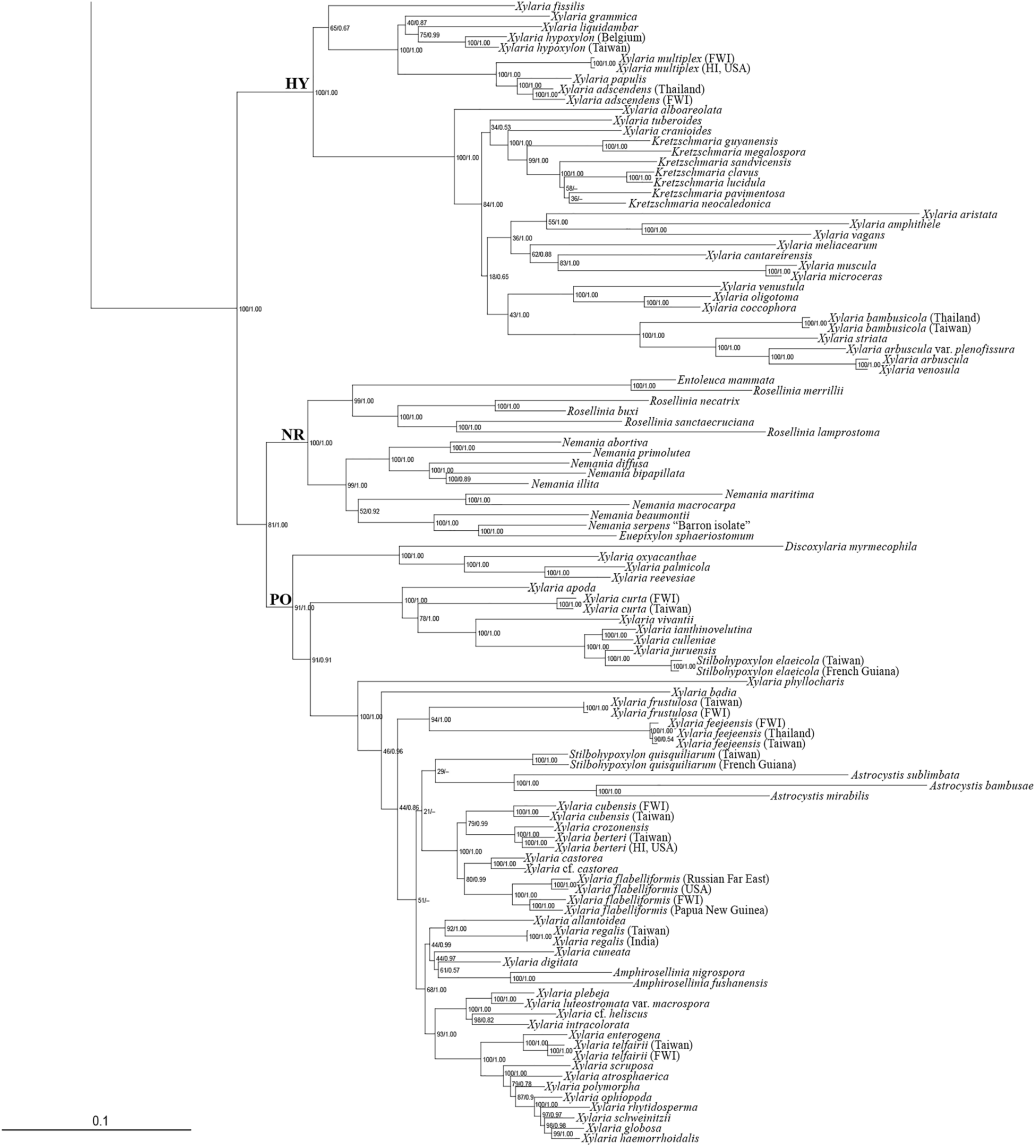
Phylogenetic tree generated by ML analysis from the RPB2-TUB-ACT dataset. The subclade of the TE clade including *X*. *furcata* and resembling species is in shade. The species newly described in the present study are in boldface. Numbers at internodes represent bootstrap values and are immediately followed by the posterior probability values greater than 50% with BI analysis. *Biscogniauxia arima* is the outgroup taxon

BI and ML analyses based on the RPB2-TUB-ACT dataset were congruent and only the ML tree is presented (Fig. 2). These trees showed that *X*. *furcata* and resembling species formed a clade with other *Xylaria* species associated with termite nests and soil. This clade corresponds with the TE clade in Hsieh et al. (2010) and U’Ren et al. (2016) and labeled as such in Fig. 2. The TE clade was divided into two major subclades: the *X*. *furcata* subclade, to which *X*. *furcata* and resembling species belonged, and the *X*. *nigripes* subclade, to which sclerotium-forming species (Ju et al. 2022) belonged. Within the *X*. *furcata* subclade, *X. furcata* and resembling species belonged to two clusters: the *X*. *furcata* cluster, grouped with *X*. *insolita* Y.-M. Ju, H.-M. Hsieh & J.-C. Chou and *X*. *ochraceostroma* Y.-M. Ju & H.-M. Hsieh in one branch, and the *X*. *hoehnelii* cluster, grouped with *X*. *intraflava* Y.-M. Ju & H.-M. Hsieh and *subintraflava* Wangsawat, Y.-M. Ju, Phosri, Whalley & Suwannasai in the other branch. The species of the *X*. *furcata* cluster, including *X*. *brevifurcata*, *X*. *furcata*, *X*. *insignifurcata*, *X*. *tenellifurcata*, *X*. *scoparia*, and *X*. *siamensis*, are characterized by half-exposed to fully exposed perithecial mounds on the stromatal surface, while those of the *X*. *hoehnelii* cluster, including *X*. *furcatula* and *X*. *hoehnelii*, are characterized by inconspicuous perithecial mounds.

## Taxonomy

### Description of *X. furcata*

**Fig. 3:**
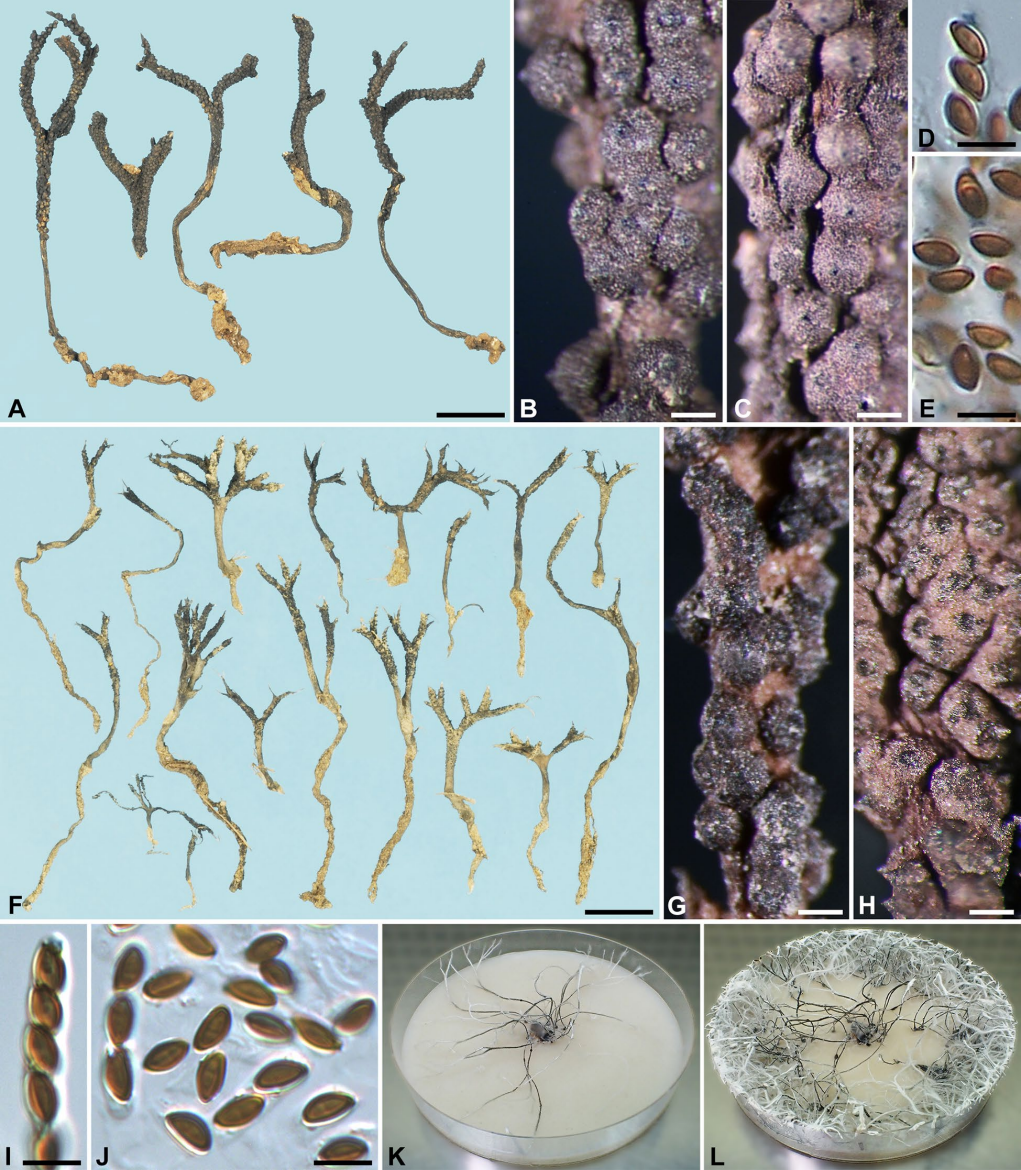
*Xylaria furcata* (**A**–**E** from neotype, **F**–**L** from epitype). **A**, **F** Stromata. **B**, **C**, **G**, **H**. Stromatal surfaces. **D**, **I** Ascal apical rings. **E**, **J** Ascospores. **K**, **L** Colony on 9-cm Petri plate containing OA at 2 and 4 wk, respectively. Bars in **F** = 1 cm; **A** = 5 mm; **B**, **C**, **G**, **H** = 0.25 mm; **D**, **E**, **I**, **J** = 5 µm

**Fig. 4:**
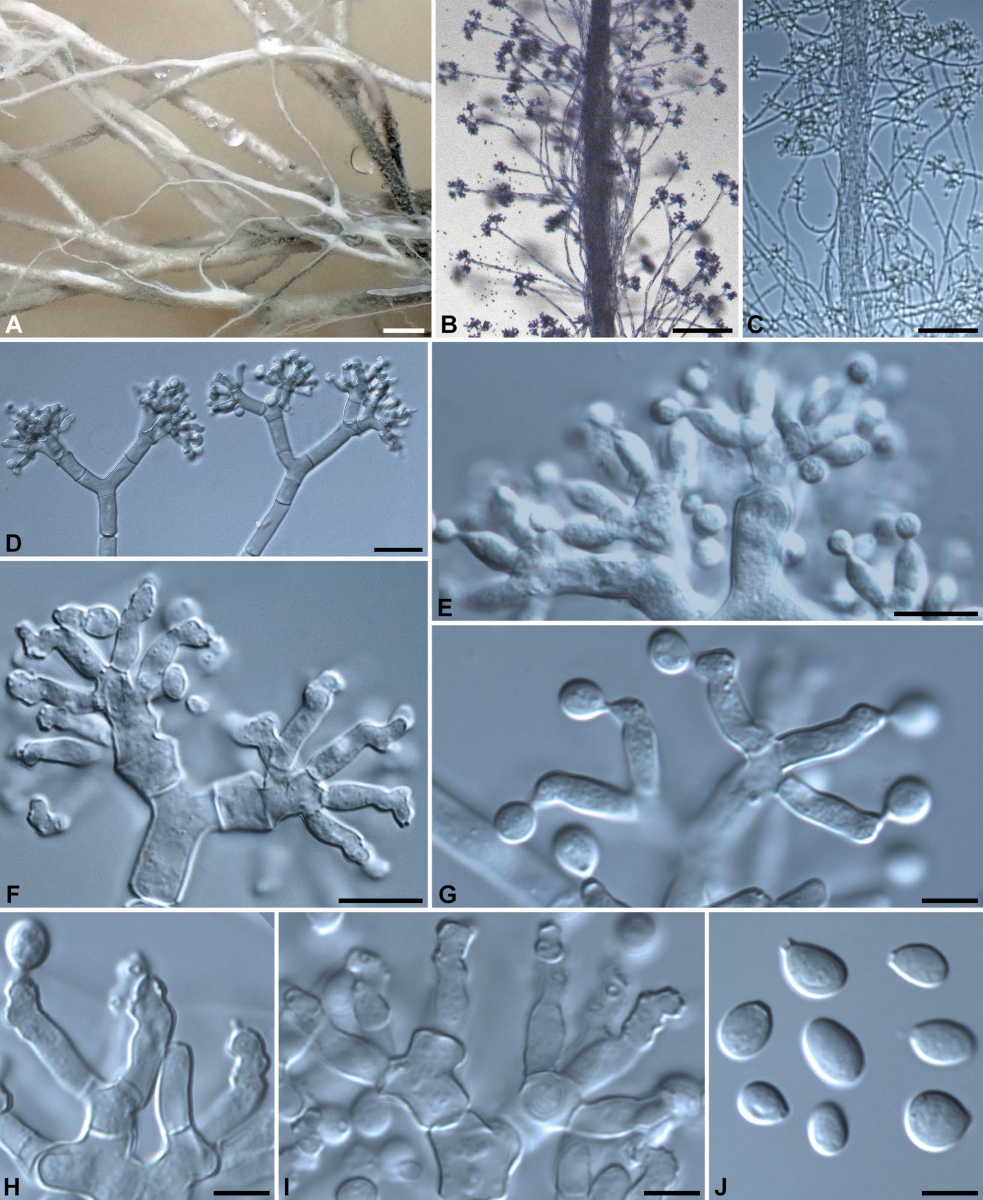
*Xylaria furcata* (from epitype). **A** Stromata produced in culture. **B**, **C** Stromatal surface bearing upright conidiophores. **D**–**F** Conidiophores. **G**–**I** Conidiogenous cells. **J** Conidia. Bars in **A** = 1 mm; **B**, **C** = 100 μm; **D** = 20 μm; **E**, **F** = 10 μm; **G**–**J** = 5 µm

***Xylaria furcata*** Fr., Nov. Act. Reg. Soc. Sci. Upsal., ser. III, 1: 128. 1851; non Schwein. ex Berk. & M. A. Curtis. Figs. 3–4

≡ *Sphaeria dichotoma* Lév., Ann. Sci. Nat., Bot., sér. III, 3: 45. 1845; non *Xylaria dichotoma* (Mont.) Fr., 1851.

≡ *Xylosphaera furcata* (Fr.) Dennis, Bull. Jardin Botan. État Brux. 31: 116. 1961.

≡ *Podosordaria furcata* (Fr.) P. M. D. Martin, J. S. African Bot. 36: 131. 1970, nom. inval. ICN Art. 41.1 (Shenzhen Code); J. S. African Bot. 42: 79. 1976.

**Typification** INDONESIA. Java, Buitenzorg, on termite nests, 1907–1908, *von Höhnel, F. A4371* (neotype [redesignated here, MycoBank Typification No. 10013825] of *X. furcata* FH ex von Höhnel herb., isoneotypes S F57929 ex Bresadola herb., FH ex von Höhnel herb. [in 3 packets], K[M] ex von Höhnel herb.); Java, Buitenzorg, on termite nests, 1908, *von Höhnel, F.*, *Rehm's Ascomyceten 1812* (isoneotype of *X. furcata* S F57927 ex Rehm herb., K[M]). TAIWAN. Tainan City, Hsin-shih District, Tan-ting, on ground of mango orchard, 28 Jul 2015, *Chou, K.-H. 104072801* (cultured from stromata *YMJ1730, YMJ1731, YMJ1732, YMJ1736, YMJ1737 & YMJ1738*) (epitype [designated here, MycoBank Typification No. 10013826] of *X. furcata* HAST 145909).

Stromata antler-like at fertile part, dichotomously branched one to three times, with acuminate apices, on a glabrous stipe, 2–3.5 cm long above ground, 1.1–1.7 cm long × 1–1.5 mm diam at fertile part; surface dark brown, becoming black, with half-exposed to fully exposed perithecial mounds but less so with close-set perithecia, lacking an outer layer, underlain with a thin, black layer ca. 10 µm thick; interior white, homogeneous, soft. Perithecia spherical, 150–250 µm diam. Ostioles conical, 60–80 µm high × 80–110 µm broad at base. Asci with eight ascospores arranged in uniseriate manner, cylindrical, 55–75 µm total length, the spore-bearing part 25–40 µm long × 3.5–4.5 µm broad, with an apical ring staining blue in Melzer's iodine reagent, inverted hat-shaped, 1–1.5 µm high × 1.5 µm broad. Ascospores light brown to brown, unicellular, short fusoid-inequilateral, slightly laterally compressed, with narrowly rounded ends, smooth, (3.5–)4–5.5(–6) × (2–)2.5–3 µm (4.7 ± 0.6 × 2.6 ± 0.3 µm, N = 80), with a straight germ slit spore-length or nearly so on the ventral side, lacking a hyaline sheath; epispore smooth.

*Cultures and anamorph:* Colonies reaching the edge of 9-cm Petri dish in 4 wk, whitish, mostly submerged, azonate, with diffuse margins. Reverse uncolored. Stromata arising copiously from the entire colonies, antler-like, branched several times, up to 5 cm long × 0.4–0.6 mm diam, white, immediately becoming black from base upwards, overlain with smoke gray pustules on the entire surface due to production of conidia. Anamorph produced on the stromatal surface. Conidiophores upright, mononematous; main axis unbranched, 200–300 × 5–8 µm, dichotomously branched two to five times on top, smooth, hyaline. Conidiogenous cells 2–3 born on each terminal short branch, initially ampulliform, becoming cylindrical, closely geniculate at upper end after producing multiple conidia in sympodial sequence, 8–12.5 × 3–4 µm, smooth, bearing terminal poroid conidial secession scars. Conidia produced holoblastically, hyaline, smooth, subglobose to ellipsoid, (4.5–)5–6.5(–7) × (3.5–)4–5(–5.5) µm (5.7 ± 0.6 × 4.4 ± 0.4 µm, N = 40), with a flattened base indicating former point of attachment to conidiogenous cell.

**Additional specimens examined** CHINA. Hainan, Ting-an, on ground, Sep 1934, *Deng, S. Q. 4219* (BPI 584600, BPI 584828, BPI 584607); Kiangsi, Tehsin, on ground, 20 May 1935, *Deng, S. Q. 9521* (BPI 584603), mixed with *X. scoparia*; Kiangsi, Tehsin, on ground, 20 May 1935, *Deng, S. Q. 9523* (BPI 584602, BPI 584829); Kiangsi, Tehsin, on ground, 20 May 1935, *Deng, S. Q. 9524* (BPI 584606); Nanking, Ling-ku-sze Woods, on ground, 28 Jun 1936, *Shen, H. N. 338* (BPI 584831); Nanking, Ling-ku-sze Woods, on ground, 13 Jun 1937, *Teng, S. C. 2743* (BPI 584601). TAIWAN. Tainan City, Hsin-shih District, Tan-ting, on ground of bamboo plantation, 1 Jul 2009, *Chou, K.-H. 98070101* (cultured from stromata *YMJ1100, YMJ1101 & YMJ1102*) (HAST 145906); Tainan City, Hsin-shih District, Tan-ting, on ground of bamboo plantation, 24 Jul 2009, *Chou, K.-H. 98072401* (cultured from stromata *YMJ1125, YMJ1126, YMJ1127, YMJ1128 & YMJ1129*) (HAST 145907); Tainan City, Hsin-shih District, Tan-ting, on ground of bamboo plantation, 3 Aug 2009, *Chou, K.-H. 98080301* (cultured from stromata *YMJ1132, YMJ1133 & YMJ1134*) (HAST 145908); Tainan City, Hsin-shih District, Tan-ting, on ground of mango orchard, 23 Jul 2009, *Chou, K.-H. 98072301* (cultured from stromata *YMJ1120, YMJ1121, YMJ1122 & YMJ1123*) (HAST 145910); Tainan City, Shen-hua District, Liu-fen-liao, from fungus comb below a *Termitomyces* mushroom, 31 Jul 2009, *Chou, K.-H. 98073103* (cultured from stromata *YMJ956 & YMJ1135*) (HAST 145947), culture only; Tainan City, Shen-hua District, Liu-fen-liao, on ground of bamboo plantation, 27 Jul 2008, *Chou, K.-H. 97072705* (cultured from stromata *YMJ978 & YMJ979*) (HAST 145911), immature, with anamorph only; Tainan City, Shen-hua District, Niu-Chuang, on ground under *Cordia dichotoma* (Boraginaceae), 15 Jul 2008, *Chou, K.-H. 97071501* (cultured from stromata *YMJ990, YMJ993 & YMJ994*) (HAST 145926), immature; Tainan City, Shen-hua District, vicinity of Asian Vegetable Research and Development Center, on termite fungus combs, 20 Jul 2006, *Chou, K.-H. 95072001* (cultured from stroma *YMJ646*) (HAST 145912), anamorph emerging from fungus combs, as *X*. sp. 4 in Hsieh et al. (2010).

**Notes** *Xylaria furcata* is characterized by its delicate, glabrous stromata that branch dichotomously one to three times. The stromata are topped with acuminate apices, and roughened with partially or fully exposed perithecial mounds on the fertile parts when fully mature. The perithecial mounds are less evident where perithecia are close-set. The mature stromatal surface is initially brown but gradually blackens with time. This species is found only in Asia thus far. Other species included in this study can be easily misidentified as *X*. *furcata* or have been confused with it. These species can be separated from *X*. *furcata* based on morphological features of the teleomorphs and anamorphs as outlined in the identification key herein.

As mentioned above, the original material of *X. furcata* seems to be missing. As a result, Rogers et al. (2005) neotypified the name with a part of a von Höhnel collection made from Java, Indonesia. Five packets of the von Höhnel collection can be located at FH and are attached on the sheet 7472 in three rows, with one, two, and two packets in the top, middle, and bottom rows, respectively. The packet on the left in the middle row was designated by Rogers et al. (2005) as the neotype for *X*. *furcata* but contains three different *Xylaria* species. The material, on which the above description of *X*. *furcata* is based, is now redesignated as the neotype while the remaining material belongs to two new species:* X*. *hoehnelii* and *X*. *robustifurcata*. The corresponding material in the packet in the top row, right packet in the middle row, and right packet in the bottom row are considered isoneotypes of *X*. *furcata* while the remaining material belongs to *X*. *hoehnelii* and/or *X*. *robustifurcata*. The left packet in the bottom row contains long stromata emerging from an incubated fungus comb shown in Plate IV of von Höhnel (1908), with an ITS sequence slightly different from that of the neotype (Fig. 1). Other parts of the von Höhnel collection were distributed as *no. 1812* of Rehm’s exsiccati *Ascomyceten*. Its duplicates at S and K contain both *X*. *furcata* and *X*. *hoehnelii*, with the *X*. *furcata* stromata considered isoneotypes while a duplicate at HBG contains *X*. *hoehnelii* and *X*. *robustifurcata* but not* X*. *furcata*.

Dennis (1961) reported *X*. *furcata* from Congo. However, in a footnote on page 118, he mentioned that the interpretation of *X*. *furcata* in Teng (1934) seemed different from his own. We have examined nine Chinese specimens that Teng identified as *X*. *furcata* from BPI and reconfirmed his identifications. The Congo specimen that Dennis (1961) considered to be *X*. *furcata* is actually *X*. *hirsuta*.

*Xylaria* cf. *furcata* reported from New Zealand by Rogers and Samuels (1986) has ascospores measuring (7.0–)7.5–8.5(–9.0) × 3.5–4.0(–4.5) μm, which are substantially larger than those of *X*. *furcata* and resembling species. It may represent an undescribed species of subg. *Pseudoxylaria*. However, it remains uncertain as to whether the New Zealand collection is associated with soil or wood buried in soil (Rogers and Samuels 1986).

### Species resembling *X. furcata*

**Fig. 5:**
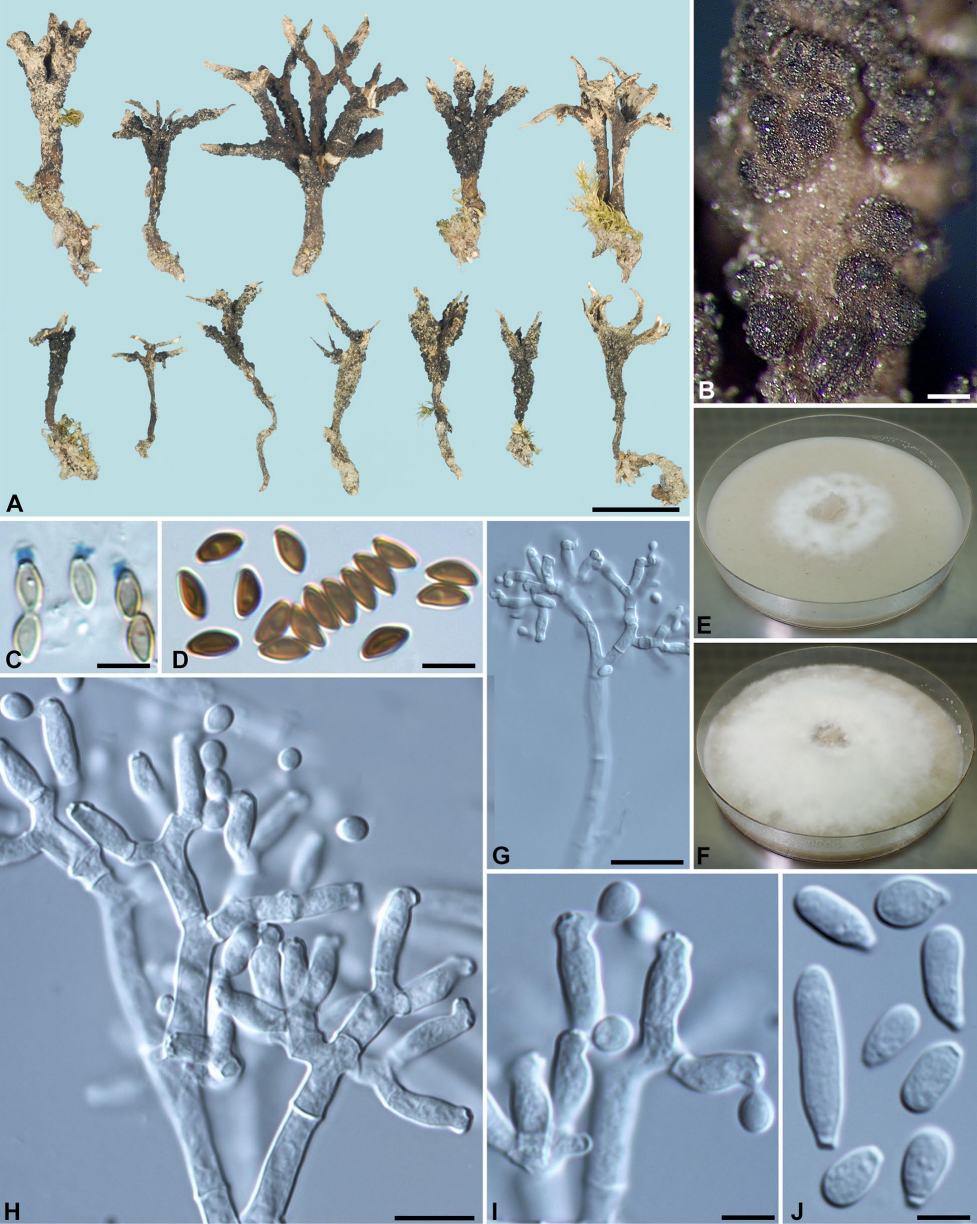
*Xylaria brevifurcata* (from holotype). **A** Stromata. **B** Stromatal surface. **C** Ascal apical rings. **D** Ascospores. **E**, **F** Colony on 9-cm Petri plate containing OA at 2 and 4 wk, respectively. **G**, **H** Conidiophores. **I** Conidiogenous cells. **J** Conidia. Bars in **A** = 5 mm; **B** = 0.25 mm; **G** = 25 μm; **H** = 10 μm; **C**, **D**, **I**, **J** = 5 µm

***Xylaria brevifurcata*** Y.-M. Ju & H.-M. Hsieh, sp. nov. Fig. 5

MycoBank MB849229.

**Typification. TAIWAN** Hua-lien County, Shou-feng Township, campus of National Dong Hwa University, on ground, 26 Jun 2011, *Chou, J.-C. 100062601* (cultured from stroma *YMJ1381*) (holotype of *X. brevifurcata* HAST 145904).

**Etymology** Referring to the short furcate stromata.

Stromata antler-like at fertile part, dichotomously branched one to several times, with acuminate apices, on a glabrous stipe, 0.9–1.5 cm long above ground, 0.4–0.6 cm long × 1.1–2.3 mm diam at fertile part; surface brown to dark brown except for black perithecial mounds, more light-colored at apices, with half-exposed to fully exposed perithecial mounds, lacking an outer layer, underlain with a thin, black layer ca. 10 µm thick; interior white, homogeneous, soft. Perithecia spherical, 150–250 µm diam. Ostioles conic-papillate, ca. 40 µm high × ca. 50 µm broad at base. Asci with eight ascospores arranged in uniseriate manner, cylindrical, 55–80 µm total length, the spore-bearing part 25–35 µm long × 3.5–4.5 µm broad, with an apical ring staining light blue in Melzer's iodine reagent, inverted hat-shaped, 1.5–2 µm high × 1.5–2 µm broad. Ascospores brown to dark brown, unicellular, short fusoid-inequilateral, slightly laterally compressed, with narrowly rounded ends, sometimes slightly pinched on one end or both ends, smooth, (4–)4.5–5(–5.5) × 2.5–3 µm (5.0 ± 0.2 × 2.6 ± 0.2 µm, N = 40), with a straight germ slit spore-length or nearly so on the ventral side, lacking a hyaline sheath; epispore smooth.

Cultures and anamorph. Colonies reaching the edge of 9-cm Petri dish in 4 wk, white, submerged, becoming cottony at places, azonate, with diffuse margins. Reverse uncolored. Stromata not produced or sporadically produced, cylindrical, tapering upwards, dichotomously branched one to several times, up to 3 cm long × 0.4–0.9 mm diam, white. Anamorph produced on the colony surface. Conidiophores upright, mononematous, 5–6 µm broad at base, dichotomously branched several times from base, smooth, hyaline. Conidiogenous cells 2–3 born on each terminal short branch, initially ampulliform, becoming cylindrical, forming one to several consecutive nodulose swellings at upper end after producing multiple conidia in sympodial sequence, 9–15 × 3.5–4 µm, smooth, bearing terminal poroid conidial secession scars. Conidia produced holoblastically, hyaline, smooth, highly variable in shape, subglobose, obovoid, ellipsoid to oblong, equilateral or slightly to significantly oblique, (4.5–)5.5–8.5(–11) × (3–)3.5–4.5(–5) µm (7.0 ± 1.6 × 4.0 ± 0.3 µm, N = 40), with a flattened base indicating former point of attachment to conidiogenous cell.

**Additional specimen examined** TAIWAN. Tainan City, Hsin-shih District, Tan-ting, on ground of mango orchard, 28 Jul 2008, *Chou, K.-H. 97072805* (cultured from stroma *YMJ986*) (HAST 145905), immature, with anamorph and developing perithecia.

**Notes** *Xylaria brevifurcata* resembles a smaller version of *X*. *furcata*, with stromata terminating into acuminate apices and perithecial mounds that are half-exposed to fully exposed. Stromata are infrequently produced in culture but remain sterile. Conidia are produced over the entire colony surface and vary greatly in shape.

**Fig. 6:**
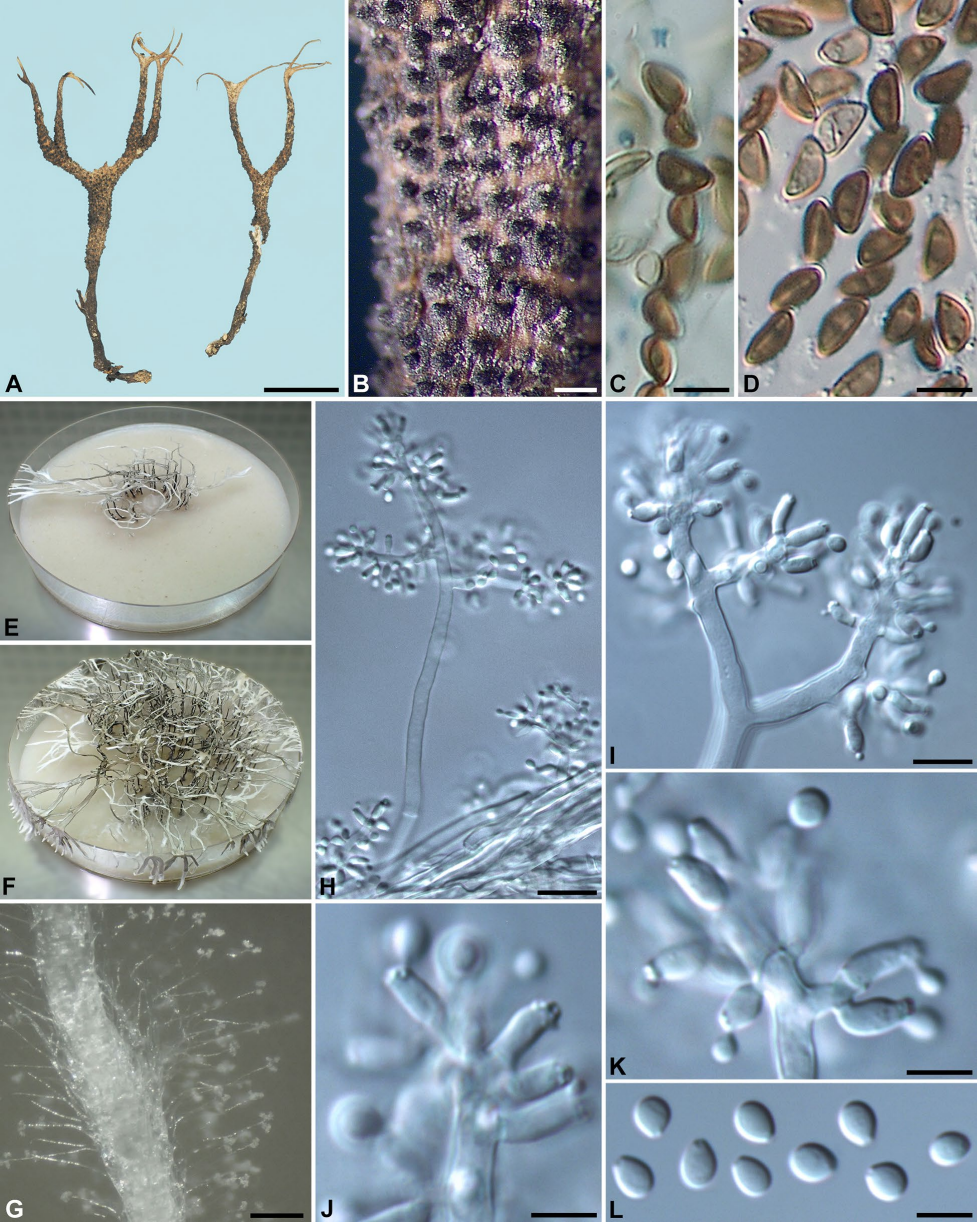
*Xylaria furcatula* (from holotype). **A** Stromata. **B** Stromatal surface. **C** Ascal apical ring. **D** Ascospores. **E**, **F** Colony on 9-cm Petri plate containing OA at 2 and 4 wk, respectively. **G** Stromatal surface bearing upright conidiophores. **H**, **I** Conidiophores. **J**, **K** Conidiogenous cells. **L** Conidia. Bars in **A** = 5 mm; **B** = 0.25 mm; **G** = 25 μm; **H** = 20 μm; **I** = 10 μm; **C**, **D**, **J**–**L** = 5 µm

***Xylaria furcatula*** Y.-M. Ju & H.-M. Hsieh, sp. nov. Fig. 6

MycoBank MB849230.

**Typification** TAIWAN. Tainan City, Hsin-shih District, Tan-ting, on ground of bamboo plantation, 1 Jul 2009, *Chou, K.-H. 98070105* (cultured from stromata *YMJ1099 & YMJ1103*) (holotype of *X. furcatula* HAST 145914).

**Etymology** Referring to the short, furcate terminal branchlets of stromata.

Stromata antler-like at fertile part, dichotomously branched one to two times and further branched at sterile apices, with delicate acuminate or forked apices, on a glabrous stipe, 2.1–2.4 cm long above ground, 1–1.2 cm long × 1.2–1.7 mm diam at fertile part; surface tan-colored to brown except for surrounding areas of ostioles, with inconspicuous perithecial mounds, lacking an outer layer, underlain with a thin layer concolorous with the surface, ca. 10 µm thick; interior white, homogeneous, soft. Perithecia spherical, 150–200 µm diam. Ostioles conical, ca. 50 µm high × ca. 50 µm broad at base. Asci with eight ascospores arranged in uniseriate manner, cylindrical, 70–90 µm total length, the spore-bearing part 35–45 µm long × 3.5–4.5 µm broad, with an apical ring staining blue in Melzer's iodine reagent, inverted hat-shaped, 1.5–2 µm high × 1.5 µm broad. Ascospores brown, unicellular, ellipsoid-inequilateral, with narrowly rounded ends, smooth, 5.5–6(–6.5) × 3–3.5 µm (5.8 ± 0.3 × 3.1 ± 0.3 µm, N = 40), with a straight germ slit spore-length or nearly so on the ventral side, lacking a hyaline sheath; epispore smooth.

Cultures and anamorph. Colonies reaching the edge of 9-cm Petri dish in 4 wk, whitish, mostly submerged, azonate, with diffuse margins. Reverse uncolored or pale tan-colored. Stromata arising copiously from the entire colonies, antler-like, branched several times, up to 5 cm long × 0.4–0.6 mm diam, white, immediately becoming black from base upwards, overlain with smoke gray pustules on the entire surface due to production of conidia. Anamorph produced on the stromatal surface. Conidiophores upright, mononematous; main axis unbranched, 100–300 × 4.5–6 µm, alternately branched several times close to top, smooth, hyaline. Conidiogenous cells formed singly or in whirls of 2–3 along each terminal branch, initially ampulliform, becoming cylindrical, slightly swollen at upper end after producing multiple conidia in sympodial sequence, 5.5–11 × 2.5–3.5 µm, smooth, bearing terminal poroid conidial secession scars. Conidia produced holoblastically, hyaline, smooth, subglobose to obovoid, 3.5–5(–6) × 3–4 µm (4.2 ± 0.6 × 3.5 ± 0.3 µm, N = 40), with a flattened base indicating former point of attachment to conidiogenous cell.

**Additional specimens examined** TAIWAN. Tainan City, Hsin-shih District, Tan-ting, on ground, 3 Jul 2017, *Chou, K.-H. 106070302* (cultured from stroma *YMJ2194*) (HAST 145913); Tainan City, Shen-hua District, Niu-Chuang, on ground under *Cordia dichotoma* (Boraginaceae), 16 Aug 2018, *Chou, K.-H. 107081602* (cultured from stromata *YMJ1916, YMJ1917 & YMJ1918*) (HAST 145915).

**Notes** Stromata of *X*. *furcatula* are characterized by having short, dichotomous sterile branchlets on top, a tan to brown surface, and inconspicuous perithecial mounds.

**Fig. 7:**
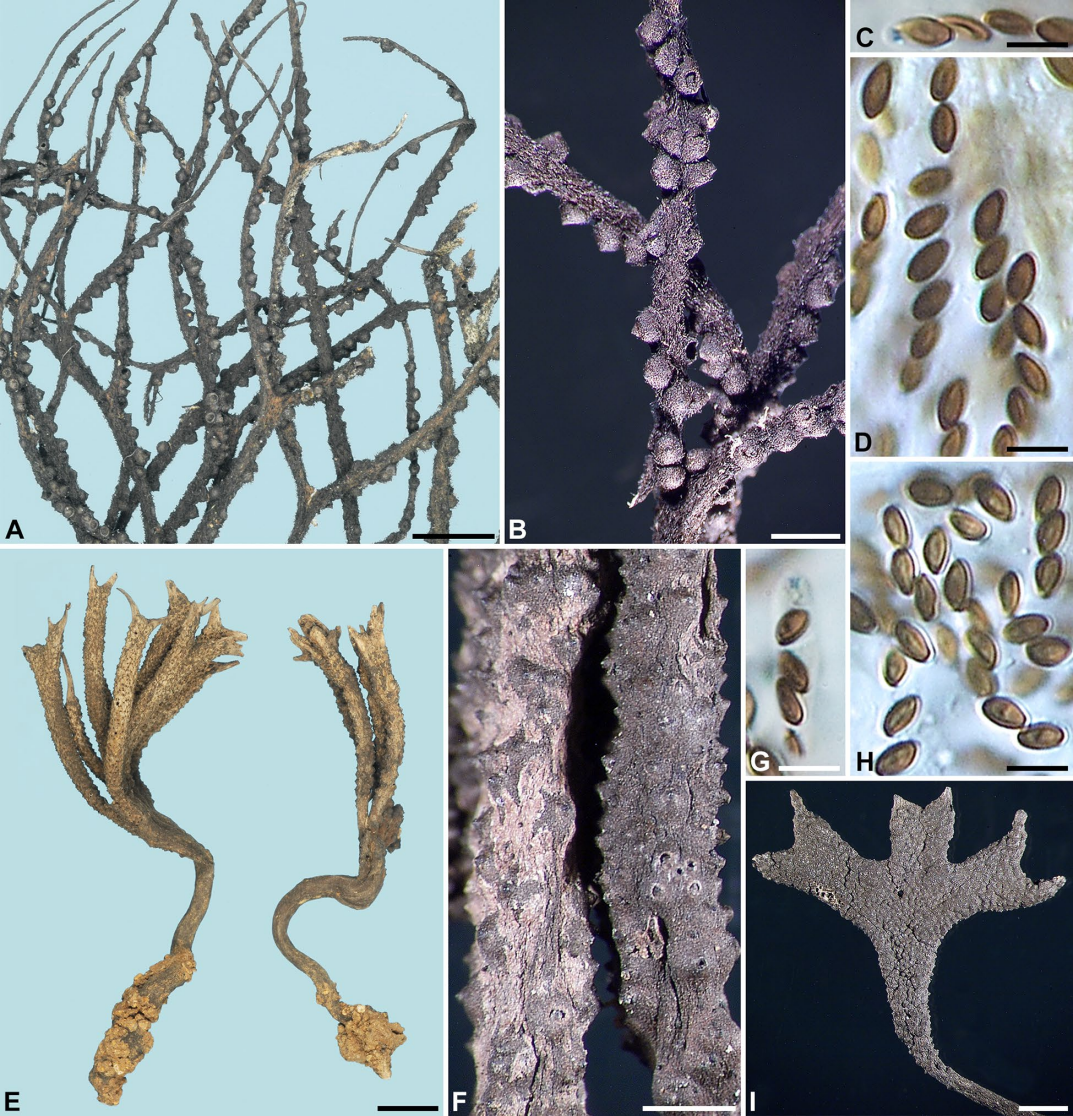
*Xylaria hirsuta* and *X*. *robustifurcata*. **A**–**D** *X*. *hirsuta* (from holotype). **A** Stroma. **B** Stromatal surface. **C** Ascal apical ring. **D** Ascospores. **E**–**I** *X*. *robustifurcata* (from holotype except for **I**, which is from the holotype of *X*. *nigripes* var. *trifida*). **E**, **I**. Stromata. **F** Stromatal surface. **G** Ascal apical ring. **H** Ascospores. Bars in **A** = 2.5 mm; **B** = 0.5 mm; **E** = 5 mm; **F**, **I** = 0.25 mm; **C**, **D**, **G**, **H** = 5 µm

***Xylaria hirsuta*** (J. D. Rogers & Y.-M. Ju) Y.-M. Ju & H.-M. Hsieh, comb. nov. Fig. 7A–D

MycoBank MB849231.

≡ *Xylaria furcata* Fr. var. *hirsuta* J. D. Rogers & Y.-M. Ju in J. D. Rogers, Y.-M. Ju & Lehmann, Mycologia 97: 919. 2005.

**Typification** DEMOCRATIC REPUBLIC OF CONGO. Yangambi, Isalowe, altitude 470 m, on soil, May 1939, *Louis, F. 14866*, as *X. furcata* Fr. by Dennis, R. W. G. (holotype of *X. furcata* var. *hirsuta* K[M] 125997).

Stromata antler-like at fertile part, highly dichotomously branched, with long acicular apices, on a tomentose stipe, 5.2 cm long above ground, 3 cm long × 1.5 mm diam at fertile part; surface blackish brown, with fully exposed perithecial mounds, overlain with a dark brown tomentum, underlain with a thin, black layer ca. 10 µm thick; interior white, homogeneous, soft. Perithecia spherical, 200–250 µm diam. Ostioles conical, ca. 60 µm high × ca. 100 µm broad at base. Asci with eight ascospores arranged in uniseriate manner, cylindrical, 45–65 µm total length, the spore-bearing part 25–35 µm long × 3–4 µm broad, with an apical ring staining light blue in Melzer's iodine reagent, inverted hat-shaped, 1–1.5 µm high × 1–1.5 µm broad. Ascospores light brown to brown, unicellular, short fusoid, inequilateral to slightly inequilateral, with narrowly rounded ends, smooth, 4–5 × 2–2.5 µm (4.5 ± 0.3 × 2.3 ± 0.2 µm, N = 40), with a straight germ slit spore-length or nearly so on the ventral side, lacking a hyaline sheath; epispore smooth.

Cultures and anamorph. Unknown.

**Notes** *Xylaria hirsuta* is known only from its holotype, which contains a stroma repeatedly dichotomously branched up to seven times. It was collected from African, featuring a hirsute stromatal surface roughened with fully exposed perithecial mounds.

**Fig. 8:**
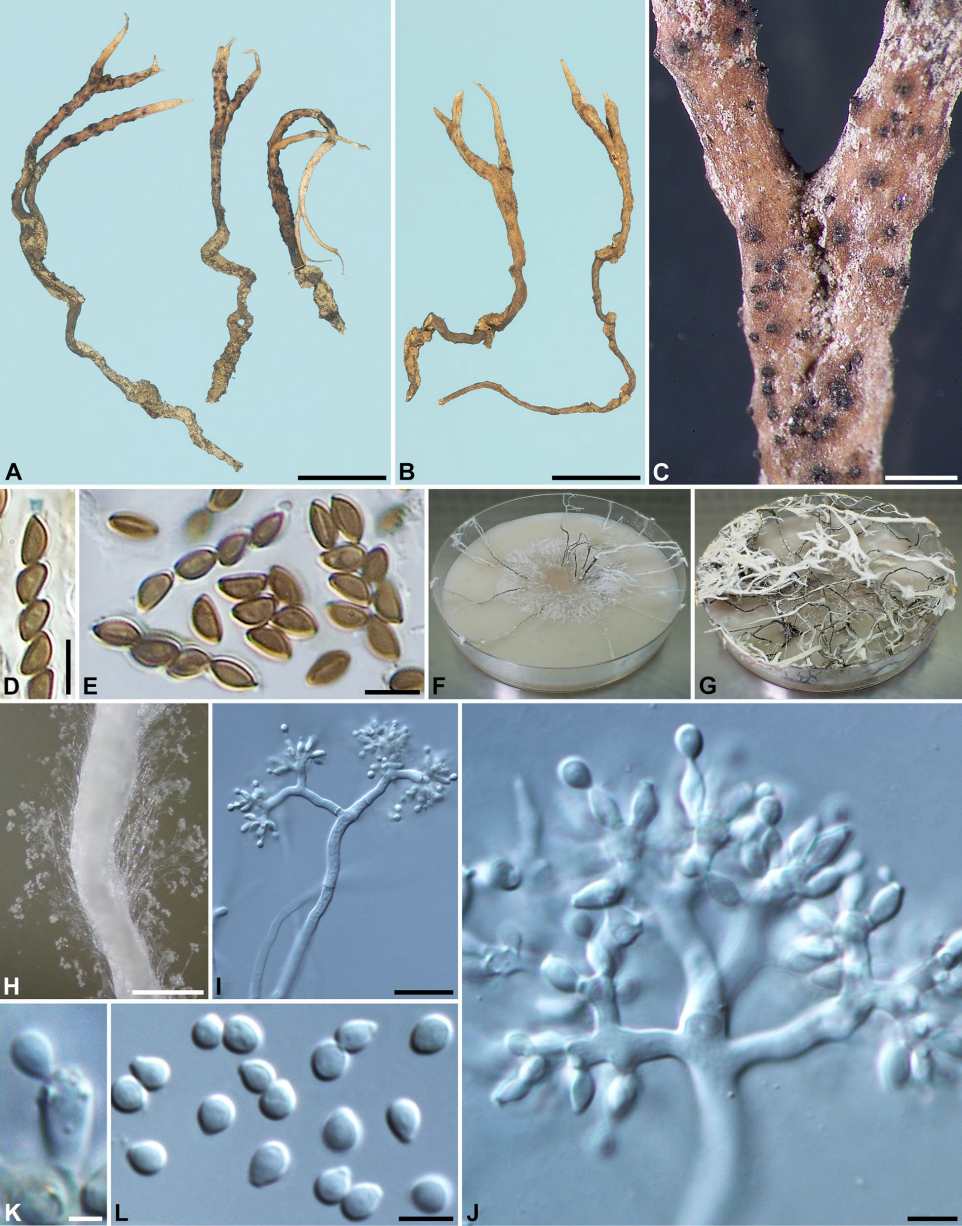
*Xylaria hoehnelii* (from holotype except for **B**, which is from von Höhnel A4371 [FH]). **A**, **B** Stromata. **C** Stromatal surface. **D** Ascal apical ring. **E** Ascospores. **F**, **G** Colony on 9-cm Petri plate containing OA at 2 and 4 wk, respectively. **H** Stromatal surface bearing upright conidiophores. **I**, **J** Conidiophores. **K** Conidiogenous cell bearing a conidium. **L** Conidia. Bars in **A**, **B** = 1 cm; **C**, **H** = 1 mm; **I** = 25 μm; **D**, **E**, **J**, **L** = 5 µm; **K** = 2 μm

***Xylaria hoehnelii*** Y.-M. Ju & H.-M. Hsieh, sp. nov. Fig. 8

MycoBank MB849233.

**Typification** TAIWAN. Tainan City, Hsin-shih District, Tan-ting, on ground of mango orchard, 20 May 2006, *Ju, Y.-M. & Hsieh, H.-M. 95052006* (cultured from stromata *YMJ642, YMJ643, YMJ644 & YMJ645*) (holotype of *X. hoehnelii* HAST 145916), as *Xylaria* sp. 1 in Hsieh et al. (2010).

**Etymology** In honor of the Austrian mycologist Franz Xaver Rudolf von Höhnel for his contribution to *X*. *furcata* and similar fungi.

Stromata antler-like at fertile part, dichotomously branched one to three times, with delicate acuminate or forked apices, on a glabrous stipe, 2–3.5 cm long above ground, 1.5–1.9 cm long × 1.5–2.1 mm diam at fertile parts; surface tan-colored to brown except for surrounding areas of ostioles, with inconspicuous perithecial mounds, lacking an outer layer, underlain with a thin layer concolorous with the surface, ca. 10 µm thick; interior white, homogeneous, soft. Perithecia spherical to obovoid, 300–400 µm diam × 400–500 µm high. Ostioles conical, 60–100 µm high × 60–80 µm broad at base. Asci with eight ascospores arranged in uniseriate manner, cylindrical, 40–65 µm total length, the spore-bearing part 25–35 µm long × 3.5–4.5 µm broad, with an apical ring staining blue in Melzer's iodine reagent, inverted hat-shaped, 1.5 µm high × 1.5 µm broad. Ascospores brown to dark brown, unicellular, ellipsoid-inequilateral, with narrowly rounded ends, smooth, 4–5 × 2.5–3 µm (4.5 ± 0.3 × 2.7 ± 0.2 µm, N = 40), with a straight germ slit spore-length or nearly so on the ventral side, lacking a hyaline sheath; epispore smooth.

Cultures and anamorph. Colonies reaching the edge of 9-cm Petri dish in 3 wk, white, submerged, immediately becoming tufted over the entire surface, azonate, with fimbriated margins. Reverse pale tan-colored. Stromata arising copiously from the entire colonies, antler-like, branched several times, up to 5 cm long × 0.4–0.9 mm diam, white, immediately becoming black from base upwards, overlain with smoke gray pustules on the entire surface due to production of conidia. Anamorph produced on the stromatal surface and hyphal strands. Conidiophores upright, mononematous; main axis unbranched, 120–200 × 4.5–6 µm, dichotomously branched two to five times on top, smooth, hyaline. Conidiogenous cells 2–3 born on each terminal short branch, initially ampulliform, hyaline, becoming cylindrical and yellowish, slightly swollen at upper end after producing multiple conidia in sympodial sequence, 5–7.5 × 2.5–3 µm, smooth, bearing terminal poroid conidial secession scars. Conidia produced holoblastically, hyaline, smooth, subglobose to obovoid, (3–)3.5–4(–4.5) × (2.5–)3–3.5 µm (3.9 ± 0.3 × 3.3 ± 0.2 µm, N = 40), with a flattened base indicating former point of attachment to conidiogenous cell.

**Additional specimens examined** INDONESIA. Java, Buitenzorg, on termite nests, 1907–1908, *von Höhnel, F. A4371* (FH ex von Höhnel herb.); Java, Buitenzorg, Bot. Garten, on termite nests, 1908, *von Höhnel, F.*, *Rehm's Ascomyceten 1812*, as *X. furcata* (S F57927a, CWU [Myc] AS716, BPI 584832, PC); Java, Mt. Salak, on ground?, *Zollinger, H.*, *Planta Javanica 857a*, as *Sphaeria gracillima* (L 910.250–1438), immature. TAIWAN. Tainan City, Hsin-shih District, Tan-ting, on ground of mango orchard, 2 Oct 2009, *Chou, K.-H. 98100204* (cultured from stroma *YMJ1159*) (HAST 145948), immature; Tainan City, Hsin-shih District, Tan-ting, on ground of mango orchard, 2 Nov 2009, *Chou, K.-H. 98111801* (cultured from stroma *YMJ1151*) (HAST 145949); Tainan City, Shen-hua District, Tung-shi, on ground, 25 Jun 2019, *Chou, K.-H. 108062508* (cultured from stromata *YMJ2178 & YMJ2179*) (HAST 145950).

**Notes** Among *X*. *furcata* and resembling species, *X. hoehnelii* and *X*. *furcatula* are the only two with a tan to brown stromatal surface and were shown to be closely related in our phylogenetic analyses (Figs. 1, 2). They also share similar colony features, conidial sizes, and *Trichoderma*-like conidiophore branching patterns. *Xylaria hoehnelii* differs from *X*. *furcatula* by having larger perithecia, 300–400 µm diam × 400–500 µm high vs. 150–200 µm diam, and smaller ascospores, 4–5 × 2.5–3 µm vs. 5.5–6(–6.5) × 3–3.5 µm. Numerous immature stromata of *X*. *hoehnelii* are commonly found fruiting from fungus combs excavated from the field in Taiwan. The holotype of *X. hoehnelii* was referred to as *Xylaria* sp. 1 in Hsieh et al. (2010).

**Fig. 9:**
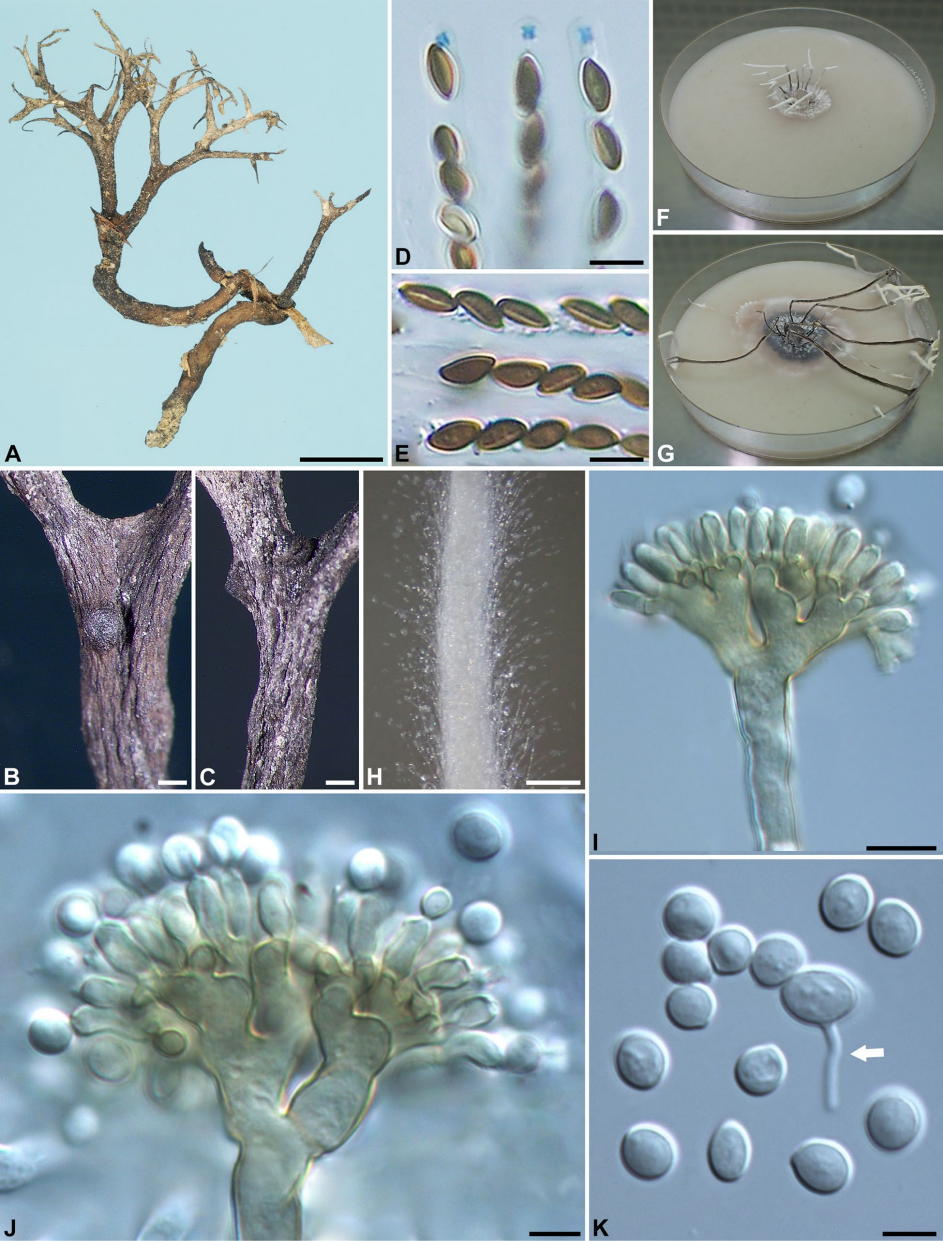
*Xylaria insignifurcata* (from holotype). **A** Stroma. **B**, **C** Stromatal surfaces. **D** Ascal apical rings. **E** Ascospores. **F**, **G** Colony on 9-cm Petri plate containing OA at 2 and 4 wk, respectively. **H** Stromatal surface bearing upright conidiophores. **I**, **J** Conidiophores. **K** Conidia; the arrow pointing towards a germinating conidium. Bars in **A** = 1 cm; **B**, **C** = 0.25 mm; **H** = 1 mm; **I** = 10 μm; **D**, **E**, **J**, **K** = 5 µm

***Xylaria insignifurcata*** Y.-M. Ju & H.-M. Hsieh, sp. nov. Fig. 9

MycoBank MB849234.

**Typification** TAIWAN. Tainan City, Hsin-shih District, Tan-ting, on ground of bamboo plantation, 12 Jul 2006, *Chou, K.-H. 95071201* (cultured from stromata *YMJ649 & YMJ650*) (holotype of *X. insignifurcata* HAST 145918), as *Xylaria* sp. 5 in Hsieh et al. (2010).

**Etymology** A remarkable furcate *Xylaria* species for its anamorph superficially resembling an *Aspergillus* species.

Stromata antler-like at fertile part, dichotomously branched one to three times, with acuminate apices, on a glabrous stipe, 5–9.6 cm long above ground, 1–2.4 cm long × 3.6–4.8 mm diam at fertile part; surface dark brown to blackish brown, with half-exposed perithecial mounds, lacking an outer layer, underlain with a thin, black layer ca. 10 µm thick; interior white, homogeneous, soft. Perithecia spherical, 300–400 µm diam. Ostioles papillate, ca. 50 µm high × ca. 120 µm broad at base. Asci with eight ascospores arranged in uniseriate manner, cylindrical, 75–95 µm total length, the spore-bearing part 30–50 µm long × 3.5–4.5 µm broad, with an apical ring staining light blue in Melzer's iodine reagent, inverted hat-shaped, 1.5 µm high × 1–1.5 µm broad. Ascospores brown to dark brown, unicellular, ellipsoid-inequilateral, with narrowly rounded ends, smooth, (4–)4.5–5.5 × 2–2.5(–3) µm (5.0 ± 0.3 × 2.4 ± 0.2 µm, N = 40), with a straight germ slit spore-length or nearly so on the ventral side, lacking a hyaline sheath; epispore smooth.

Cultures and anamorph. Colonies reaching the edge of 9-cm Petri dish in 8 wk, white initially, becoming blackish immediately behind the growing margins, velvety, appressed, zonate, with diffuse margins. Reverse tan-colored. Stromata arising from concentric zones and elongating much farther beyond the colonies, cylindrical, tapering upwards, unbranched or branched once or twice closer to the apex, up to 6 cm long × 0.9–1.2 mm diam, white, immediately becoming black from base upwards, overlain with pale mouse gray pustules on the entire surface due to production of conidia. Anamorph produced on the stromatal surface. Conidiophores upright, mononematous; main axis unbranched or branched once close to top, 150–250 × 7–9 µm, dichotomously branched three to four times in short intervals on top, smooth, yellowish to light brown. Conidiogenous cells 2–3 born on each terminal short branch, initially ampulliform, becoming cylindrical, unswollen at upper end after producing multiple conidia in sympodial sequence, 5–7.5 × 2.5–3 µm, smooth, bearing terminal poroid conidial secession scars. Conidia produced holoblastically, hyaline, smooth, subglobose to obovoid, (4.5–)5–6(–7) × (4–)4.5–5(–5.5) µm (5.5 ± 0.6 × 4.7 ± 0.3 µm, N = 40), with a flattened base indicating former point of attachment to conidiogenous cell.

**Additional specimens examined** TAIWAN. Tainan City, Shen-hua District, Liu-fen-liao, on ground of bamboo plantation, 3 Jul 2006, *Chou, K.-H. 95070301* (HAST 145919), immature, with anamorph and developing perithecia; Tainan City, Shen-hua District, Liu-fen-liao, on ground of bamboo plantation, 28 Jun 2010, *Chou, K.-H. 99062803* (cultured from stroma *YMJ1198*) (HAST 145920), immature, with anamorph only.

**Notes** Among *X*. *furcata* and resembling species, *X. insignifurcata* stands out for its larger stromata and *Aspergillus*-like conidiophores. It was referred to as *Xylaria* sp. 5 in Hsieh et al. (2010). The teleomorph of this species was found in only one stroma, where the perithecia are sparse and scattered.

***Xylaria robustifurcata*** Y.-M. Ju & H.-M. Hsieh, sp. nov. Fig.  7E–I

 = *Xylaria nigripes* (Klotzsch) M. C. Cooke var. *trifida* Pat., J. Bot. (Morot) 5: 317. 1891.

MycoBank MB849235.

**Typification** INDONESIA. Java, Buitenzorg, on termite nests, 1907–1908, *von Höhnel, F. A4371*, as *X. furcata* Fr. (holotype of *X. robustifurcata* FH ex von Höhnel herb., isotypes S F57928 ex Rehm herb., S F57929a ex Bresadola herb.); Java, Buitenzorg, Bot. Garten, on termite nests, 1908, *von Höhnel, F.*, *Rehm's Ascomyceten 1812*, as *X. furcata* (isotypes of *X. robustifurcata* HBG, PC).

**Etymology** Referring to the robust furcate stromata.

Stromata antler-like at fertile part, dichotomously branched several times, with palmate apices, on a glabrous stipe, 3–4 cm long above ground, 1–2.4 cm long × 2–2.6 mm diam at fertile part; surface grayish brown, becoming blackish when outer layer worn off, with inconspicuous to conspicuous perithecial mounds, overlain with a grayish brown outer layer gradually worn off during maturation, underlain with a thin, black layer ca. 10 µm thick; interior white, with a black core, coriaceous. Perithecia spherical, 300–400 µm diam. Ostioles conical, 80–100 µm high × 90–110 µm broad at base. Asci with eight ascospores arranged in uniseriate manner, cylindrical, 60–85 µm total length, the spore-bearing part 30–40 µm long × 4–4.5 µm broad, with an apical ring staining light blue in Melzer's iodine reagent, inverted hat-shaped, 1 µm high × 1 µm broad. Ascospores light brown to brown, unicellular, ellipsoid-inequilateral, with narrowly to broadly rounded ends, smooth, (3.5–)4–4.5 × 2–2.5 µm (4.1 ± 0.2 × 2.3 ± 0.2 µm, N = 40), with a straight germ slit spore-length or nearly so on the ventral side, lacking a hyaline sheath; epispore smooth.

Cultures and anamorph. Unknown.

**Additional specimen examined** VIETNAM. Tonkin, Ke'so', vieilles souches, *Bon*, as *X. escharoidea* var. *trifida* (holotype of *X. nigripes* var. *trifida* PC 0086053).

**Notes** *Xylaria robustifurcata* is characterized by its light brown outer stromatal layer that persists into maturity and short sterile stromatal apices. Unlike *X*. *furcata*, its stromata are more robust, and its perithecia are largely immersed, resulting in inconspicuous to conspicuous but not exposed mounds on the stromatal surface. As already mentioned in the notes for *X*. *furcata*, *X. robustifurcata* is one of the three *Xylaria* species found in a von Höhnel collection of *X*. *furcata* from Java. In addition to the five packets stored in FH, the von Höhnel collection was also distributed as Rehm’s exsiccata *Ascomyceten 1812*.

The holotype packet of *X. nigripes* var. *trifida*, filed as *X*. *escharoidea* var. *trifida* in PC, contains a stroma, which undoubtedly represents one of the two stromata depicted in Patouillard (1891) and is thus considered the holotype. The stroma is overmature, having the outer layer no longer detectable.

**Fig. 10:**
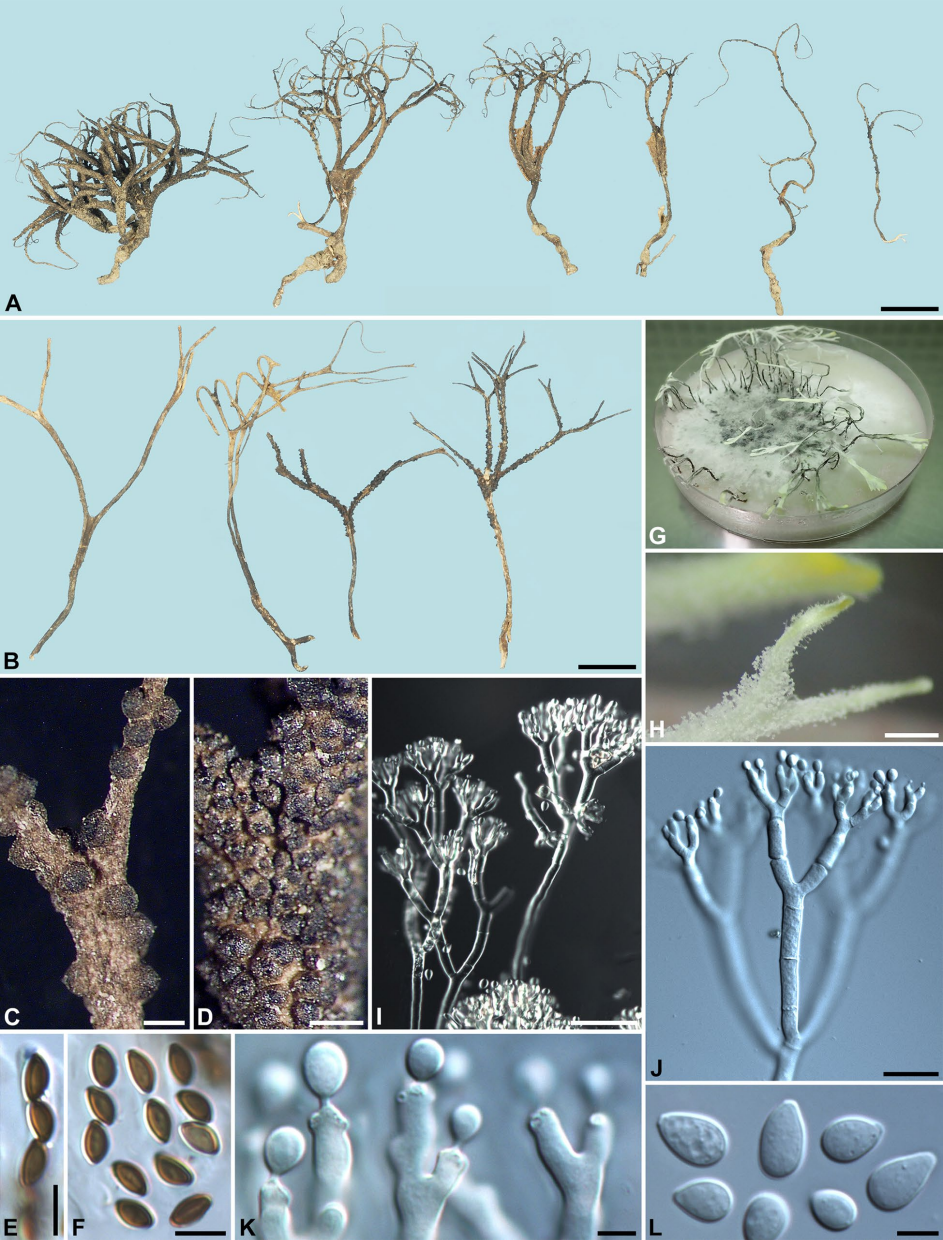
*Xylaria scoparia* (from epitype except for **B**, which is from holotype). **A**, **B** Stroma. **C**, **D** Stromatal surfaces. **E**. Ascal apical ring. **F** Ascospores. **G** Colony on 9-cm Petri plate containing OA at 8 wk. **H**, **I**. Stromatal surface bearing upright conidiophores. **J** Conidiophore. **K** Conidiogenous cells bearing conidia. **L** Conidia. Bars in **A**, **B** = 5 mm; **C**, **D** = 0.25 mm; **H** = 1 mm; **I** = 50 μm; **J** = 25 μm; **E**, **F**, **K**, **L** = 5 µm

***Xylaria scoparia*** Pat., J. Bot. (Morot) 5:318. 1891. Fig. 10

**Typification** VIETNAM. Tonkin, on ground, *Bon, H.* (lectotype [designated here, MycoBank Typification No. 10013827] of *X. scoparia* FH ex Patouillard herb.); Tonkin, Ha Noi, on ground, 1890, *Bon, H. 4399* (syntype of *X. scoparia* FH ex Patouillard herb., isosyntype PC), immature; Tonkin, Ke'so', on ground, 28 Jul 1890, *Bon, H. 4470* (syntype of *X. scoparia* FH ex Patouillard herb., isosyntypes PC [in 2 packets]), immature. TAIWAN. Tainan City, Shen-hua District, Liu-fen-liao, on ground of bamboo plantation, 3 Aug 2011, *Chou, K.-H. 100080301* (cultured from stroma *YMJ1435*) [epitype (designated here, MycoBank Typification No. 10013828) of *X. scoparia* HAST 145925].

Stromata antler-like at fertile part, dichotomously branched one to, more frequently, many times, with long acicular and curly apices, on a glabrous stipe, 2.4–4.2 cm long above ground, 1–1.5 cm long × 0.7–3.3 mm diam at fertile part; surface brown to dark brown except for black perithecial mounds, with half-exposed to fully exposed perithecial mounds, lacking an outer layer, underlain with a thin, black layer ca. 10 µm thick; interior white or pale yellow, homogeneous, soft. Perithecia spherical, 150–200 µm diam. Ostioles depressed-conical, ca. 40 µm high × ca. 80 µm broad at base. Asci with eight ascospores arranged in uniseriate manner, cylindrical, 60–80 µm total length, the spore-bearing part 30–40 µm long × 3.5–4.5 µm broad, with an apical ring staining blue in Melzer's iodine reagent, inverted hat-shaped, 1.5 µm high × 1–1.5 µm broad. Ascospores brown to dark brown, unicellular, short fusoid-inequilateral, slightly laterally compressed, with narrowly rounded ends, smooth, 4.5–5 × 2.5–3 µm (4.9 ± 0.3 × 2.6 ± 0.2 µm, N = 40), with a straight germ slit spore-length or nearly so on the ventral side, lacking a hyaline sheath; epispore smooth.

Cultures and anamorph. Colonies reaching the edge of 9-cm Petri dish in 8 week, whitish, mostly submerged, azonate, with diffuse margins. Reverse uncolored or pale tan-colored. Stromata arising copiously from the entire colonies, antler-like, branched several times, up to 5 cm long × 0.7–1.3 mm diam, white but frequently yellow on apices, immediately becoming black from base upwards, overlain with pale mouse gray pustules on the entire surface due to production of conidia. Anamorph produced on the stromatal surface. Conidiophores upright, mononematous; main axis unbranched, 150–300 × 6–9 µm, dichotomously branched two to five times on top, smooth, hyaline. Conidiogenous cells 2–3 born on each terminal short branch, initially ampulliform, becoming cylindrical, closely geniculate at upper end after producing multiple conidia in sympodial sequence, 8–18 × 3.5–4.5 µm, smooth, bearing terminal poroid conidial secession scars. Conidia produced holoblastically, hyaline, smooth, subglobose to obovoid, (5–)6.5–8.5(–10) × (4.5–)5–6(–6.5) µm (7.5 ± 1.1 × 5.3 ± 0.5 µm, N = 40), with a flattened base indicating former point of attachment to conidiogenous cell.

**Additional specimens examined** CHINA. Kiangsi, on ground, 20 May 1935, *Deng, S. Q. 4106*, as *X. furcata* (UPS). MALAYSIA. Malay Peninsula, Pulau Penang, Waterfall Gardens, on termite nests, 23 Jan 1920, *Noor, M. 15366*, as *X. furcata* (BPI 584609), immature; Malay Peninsula, Pulau Penang, Waterfall Gardens, on termite nests, 23 Jan 1920, *Noor, M. 5625*, as *X. furcata* (BPI 714719 ex Lloyd herb. 10425, BPI 584610 ex Reinking herb.), immature. PHILIPPINES. Leyte, Palo, on ground, Jan 1906, *Elmer, A. D. E. 7233* (BPI 584827 ex Bresadola herb.), immature. SINGAPORE. on ground, 27 Dec 1919, *Chipp, T. F.*, as *X. furcata* (BPI 713933 ex Lloyd herb. 11865), immature. SRI LANKA. on ground, *Petch, T. 21*, as *X. furcata* (BPI 713932 ex Lloyd herb. 11858). TAIWAN. Hua-lien County, Shou-feng Township, campus of National Dong Hwa University, on ground, 20 Jul 2011, *Chou, J.-C. 100072002* (cultured from stromata *YMJ1427, YMJ1428 & YMJ1429*) (HAST 145921); Hua-lien County, Shou-feng Township, campus of National Dong Hwa University, on ground, 3 Sep 2011, *Chou, J.-C. 100092103* (cultured from stromata *YMJ1423 & YMJ1424*) (HAST 145922), immature; Tainan City, Hsin-shih District, Tan-ting, on ground of mango orchard, 18 Jul 2016, *Chou, K.-H. 105071801* (cultured from stroma *YMJ2177*) (HAST 145951); Tainan City, Hsin-shih District, Tan-ting, on ground of mango orchard, 28 Jul 2008, *Chou, K.-H. 97072802* (cultured from stromata *YMJ982 & YMJ983*) (HAST 145923), immature, with anamorph and developing perithecia; Tainan City, Hsin-shih District, Tan-ting, on ground of mango orchard, 2 Oct 2009, *Chou, K.-H. 98100203* (cultured from stroma *YMJ1158*) (HAST 145924); Tainan City, Shen-hua District, Niu-Chuang, on ground under *Cordia dichotoma* (Boraginaceae), 26 Jul 2008, *Chou, K.-H. 97072601* (HAST 145927); Tainan City, Shen-hua District, Niu-Chuang, on ground under *Cordia dichotoma* (Boraginaceae), 15 Jul 2008, *Chou, K.-H. 97071504* (cultured from stromata *YMJ958, YMJ959, YMJ960, YMJ989, YMJ991 & YMJ992*) (HAST 145952), immature.

**Notes** *Xylaria scoparia* was considered a synonym of *X*. *furcata* by Rogers et al. (2005). However, our molecular phylogenetic analyses (Figs. 1, 2) confirmed that both species are distinct. Compared to *X*. *furcata*, *X*. *scoparia* has stromata that are frequently repeatedly branched many times, long acicular and curly stromatal apices, and darker ascospores, thus resembling *X*. *tenellifurcata*, which differs mainly by having smaller conidia and lacking the yellow apices frequently found on immature stromata produced in cultures of *X*. *scoparia* (Fig. 10H).

***Xylaria siamensis*** Wangsawat, Y.-M. Ju, Phosri, Whalley & Suwannasai, Biology (Basel) 10: 575: 21. 2021.

For descriptions of the teleomorph, cultures, and anamorph, see Wangsawat et al. (2021) where illustrations of stromata, ascus, ascospores, colony, conidiophores, and conidia are also provided. *Xylaria siamensis* is characterized by the following features: stromata antler-like at fertile part, dichotomously branched one to many times, with long acicular and curly apices, on a glabrous stipe, 2–6.7 cm long above ground, 0.3–1 cm long × 0.2–1.5 mm diam at fertile part; stromatal surface dark brick to black, with conspicuous to half-exposed perithecial mounds, lacking an outer layer, with a white, soft interior; perithecia spherical, 200–400 µm broad, with a coarsely conic-papillate ostiole ≤ 100 µm broad at base; ascospores brown to dark brown, ellipsoid-inequilateral, with narrowly rounded ends, smooth, 5–6 × 2.5–3.5 µm, with a straight germ slit spore-length on the ventral side.

**Notes** *Xylaria siamensis* is currently known only from Thailand. It resembles *X*. *scoparia* and *X*. *tenellifurcata* in having stromata with exposed perithecial mounds on the surface and long acicular and curly apices. However, it differs from the latter two species mainly by its coarsely conic-papillate ostioles and slightly longer ascospores. Wangsawat et al. (2021) obtained cultures, where stromata and anamorph were not produced. The anamorph found on the surface of young stromata from nature is much like that of *X*. *furcata*, except for having smaller conidia 4–4.5(–5) × 3–4 µm vs. (4.5–)5–6.5(–7) × (3.5–)4–5(–5.5) µm in *X. furcata*.

**Fig. 11:**
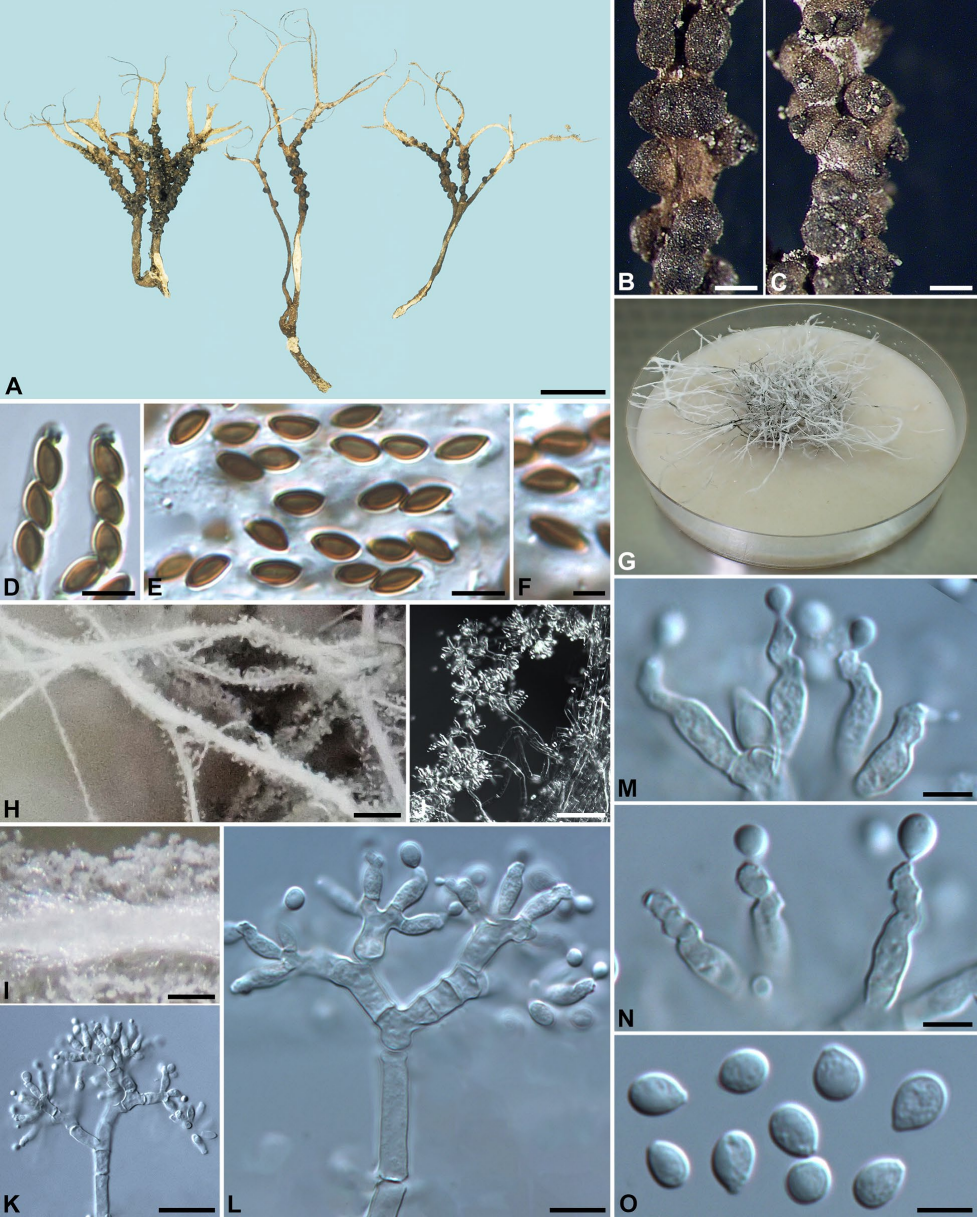
*Xylaria tenellifurcata* (from holotype). **A** Stromata. **B**, **C** Stromatal surfaces. **D** Ascal apical rings. **E**, **F** Ascospores; ascospores showing a straight to slightly sigmoid germ slit in (**F**). **G** Colony on 9-cm Petri plate containing OA at 4 wk. **H** Stromata produced in culture. **I**, **J**. Stromatal surface bearing upright conidiophores. **K**, **L** Conidiophore. **M**, **N** Conidiogenous cells bearing conidia. **O** Conidia. Bars in **A** = 5 mm; **B**, **C** = 0.25 mm; **H** = 1 mm; **I** = 0.5 mm; **J** = 50 μm; **K** = 25 μm; **L** = 10 μm; **D**, **E**, **M**–**O** = 5 µm; **F** = 2.5 μm

***Xylaria tenellifurcata*** Y.-M. Ju & H.-M. Hsieh, sp. nov. Fig. 11

MycoBank MB849236.

**Typification** TAIWAN. Tainan City, Hsin-shih District, Tan-ting, on ground of bamboo plantation, 20 May 2009, *Chou, K.-H. 98052001* (cultured from stroma *YMJ1070*) (holotype of *X. tenellifurcata* HAST 145928).

**Etymology** Referring to the delicate furcate stromata.

Stromata antler-like at fertile part, dichotomously branched one to many times, with long acicular and curly apices, on a glabrous stipe, 1.9–3 cm long above ground, 0.6–1 cm long × 1.2–1.6 mm diam at fertile part; surface brown to dark brown except for black perithecial mounds, with 2/3-exposed to fully exposed perithecial mounds, lacking an outer layer, underlain with an extremely thin, black layer less than 10 µm thick; interior white, homogeneous, soft. Perithecia spherical, 200–250 µm diam. Ostioles depressed-conical, ca. 40 µm high × ca. 80 µm broad at base. Asci with eight ascospores arranged in uniseriate manner, cylindrical, 55–70 µm total length, the spore-bearing part 35–40 µm long × 3.5–4.5 µm broad, with an apical ring staining blue in Melzer's iodine reagent, inverted hat-shaped, 1.5 µm high × 1–1.5 µm broad. Ascospores brown to dark brown, unicellular, short fusoid-inequilateral, slightly laterally compressed, with narrowly rounded ends, smooth, 4.5–5(–5.5) × 2.5–3 µm (5.0 ± 0.2 × 2.7 ± 0.2 µm, N = 40), with a straight to slightly sigmoid germ slit spore-length or nearly so on the ventral side, lacking a hyaline sheath; epispore smooth.

Cultures and anamorph. Colonies reaching the edge of 9-cm Petri dish in 6 wk, whitish, mostly submerged, azonate, with diffuse margins. Reverse pale tan-colored. Stromata arising copiously from the entire colonies, antler-like, branched several times, up to 5 cm long × 0.4–0.6 mm diam, white, immediately becoming black from base upwards, overlain with pale mouse gray pustules on the entire surface due to production of conidia. Anamorph produced on the stromatal surface. Conidiophores upright, mononematous; main axis unbranched, 100–200 × 6–7 µm, dichotomously branched two to five times on top, smooth, hyaline. Conidiogenous cells 2–3 born on each terminal short branch, initially ampulliform, becoming cylindrical, forming one to several consecutive nodulose swellings at upper end after producing multiple conidia in sympodial sequence, 7.5–13 × 3.5–4.5 µm, smooth, bearing terminal poroid conidial secession scars. Conidia produced holoblastically, hyaline, smooth, subglobose to obovoid, 4.5–5.5 × 3.5–4.5(–5) µm (5.0 ± 0.3 × 4.0 ± 0.3 µm, N = 40), with a flattened base indicating former point of attachment to conidiogenous cell.

**Notes** *Xylaria tenellifurcata* is similar to a smaller version of *X*. *scoparia*. Both species have long acicular and curly stromatal apices but can be distinguished primarily by the smaller conidia in *X*. *tenellifurcata* and by the yellow apices frequently found on immature stromata produced in cultures of *X*. *scoparia* (Fig. 10H). *Xylaria siamensis* also has long acicular and curly stromatal apices but differs in having coarsely conic-papillate ostioles and slightly longer ascospores. Multi-locus sequence data confirms these three species are distinct.

### Identification key to *X. furcata* and resembling species

1. Stromata black or tan-colored at center, overlain with a grayish brown outer layer, becoming dull blackish when outer layer worn off; ascospores ellipsoid-inequilateral, (3.5–)4–4.5 × 2–2.5 µm…*X*. *robustifurcata*
2. Stromata with a homogenous interior, lacking an outer layer…2
3. Perithecia immersed, with inconspicuous perithecial mounds…3

2ʹ. Perithecial mounds conspicuous, half-exposed to fully exposed…4

3. Stromata 1.2–1.7 mm diam at fertile part; ascospores 5.5–6(–6.5) × 3–3.5 µm…*X*. *furcatula*

3ʹ. Stromata 1.5–2.1 mm diam at fertile parts; ascospores 4–5 × 2.5–3 µm…*X*. *hoehnelii*

4. Stromatal surface hairy on fertile parts; ascospores 4–5 × 2–2.5 µm…*X. hirsuta*

4ʹ. Stromatal surface glabrous on fertile parts…5

5. Stromata 5–9.6 cm long above ground, 3.6–4.8 mm diam at fertile part; ascospores ellipsoid-inequilateral (4–)4.5–5.5 × 2–2.5(–3) µm; conidiophores dichotomously branched 3–4 times in short intervals on top…*X*. *insignifurcata*

5ʹ. Stromata mostly less than 5 cm long above ground, less than 3.5 mm diam; conidiophores not branched in short intervals on top…6

6. Stromata terminating into acuminate apices…7

6ʹ. Stromata terminating into long acicular and curly apices…8

7. Stromata 2–3.5 cm long above ground; ascospores (3.5–)4–5.5(–6) × (2–)2.5–3 µm; conidia subglobose to ellipsoid, (4.5–)5–6.5(–7) × (3.5–)4–5(–5.5) µm…*X. furcata*

7ʹ. Stromata 0.9–1.5 cm long above ground; ascospores (4–)4.5–5(–5.5) × 2.5–3 µm; conidia highly variable in shape, subglobose, obovoid, ellipsoid to oblong, equilateral or slightly to significantly oblique, (4.5–)5.5–8.5(–11) × (3–)3.5–4.5(–5) µm…*X*. *brevifurcata*

8. Ostioles coarsely conic-papillate; ascospores 5–6 × 2.5–3.5 μm; conidia 4–4.6(–5) × 3–4 μm…*X*. *siamensis*

8ʹ. Ostioles depressed-conical; ascospores slightly shorter…9

9. Ascospores 4.5–5 × 2.5–3 µm; stromata produced in culture frequently with yellow apices; conidia (5–)6.5–8.5(–10) × (4.5–)5–6(–6.5) µm…*X*. *scoparia*

9ʹ. Ascospores 4.5–5(–5.5) × 2.5–3 µm; stromata produced in culture with white apices; conidia 4.5–5.5 × 3.5–4.5(–5) µm…*X*. *tenellifurcata*

## Discussion

Species diversity of *Xylaria* subg. *Pseudoxylaria* had not been much investigated until the study of Rogers et al. (2005), where 11 taxa were recognized. The species number has since been greatly enriched, with 24 more taxa added to the subgenus (Chou et al. 2017; Hsieh et al. 2020, 2022; Ju and Hsieh 2007; Ju et al. 2011, 2022; Kim et al. 2016; Wangsawat et al. 2021). *Xylaria* collections from termite nests with delicate, dichotomously branched stromata are commonly identified as *X*. *furcata*, but our study showed that multiple species can be delimited by morphological features of teleomorphs and anamorphs as well as sequences of multiple DNA loci.

### Species delimitation among *X. furcata* and resembling species

*Xylaria furcata* and resembling species are all antler-like and share similar sizes of asci, ascal apical rings, and ascospores. *Xylaria robustifurcata* can be readily separated from the other species by having a dark stromatal core and an outer splitting layer overlying the stromatal surface. Its perithecia are entirely immersed within stromatal tissue, a feature also found in *X*. *furcatula* and *X*. *hoehnelii*. Other species, including *X*. *furcata*, *X*. *brevifurcata*, *X*. *hirsuta*, *X*. *insignifurcata*, *X*. *scoparia*, *X*. *siamensis*, and *X*. *tenellifurcata*, normally have conspicuously exposed perithecial mounds and were shown more closely related among themselves (Figs. 1, 2). All of the species are glabrous on the stromatal surface and have geographic distributions in Asia except for *X. hirsuta*, which has a hirsute stromatal surface and is known only from Africa. The average size of ascospores among the species ranges from 4.1 to 5.8 µm in length and 2.3 to 3.1 µm in width, with little variation between species. On the other hand, conidial sizes vary greatly, with averages ranging from 3.9 to 7.5 µm in length and 3.3 to 5.3 µm in width. This makes conidial sizes useful for distinguishing between species that are difficult to separate by their teleomorphs, such as *X. furcata* and *X. brevifurcata* or *X. scoparia* and *X. tenellifurcata*.

### Anamorph of *X. furcata*

Petch (1913) was probably the first to attempt to link an anamorphic *Xylaria* to *X. furcata*, describing *Botrytis*-like conidiophores formed on the stromata. These conidiophores are highly branched and terminate into a lobed head on each branch. A whorl of flask-shaped conidiogenous cells is born on these heads. The conidia were reported as being born in catenulate chains on the flask-like conidiogenous cells. This is exceptional for a *Xylaria* anamorph, where conidia are normally produced holoblastically in sympodial sequence. It should be noted that Petch (1906) may have already described and illustrated the *Botrytis*-like conidiophores as *Piptocephalis*-like from stromata induced from fungus combs kept in moisture chambers but did not specifically link it to *X*. *furcata*.

Dixon (1965) observed an anamorphic *Xylaria* species from fungus combs of an African termite and considered it the anamorph of *X. furcata*. This fungus produces two conidia in acropetal chain on each conidiogenous cell and released conidia ballistically. It was subsequently named as *Padixonia bispora* Subram. by Subramanian (1972). Dixon (1985) later considered the fungus not the anamorph of *X. furcata* but a new *Xylaria* species.

These earlier attempts to connect the teleomorph and anamorph of *X*. *furcata* were equivocal and inconsistent likely because their studied materials were not actually *X*. *furcata*. Those observations of Petch (1906, 1913) were based on *Xylaria* stromata emerging from fungus combs, where a variety of other fungi coexist with the *Xylaria* stromata sympatrically and some of them produce conidia in catenulate chains (personal observation of Y-MJ).

The anamorphs of the species being studied have poroid conidial secession scars on top of swollen conidiogenous cells after producing multiple conidia. This differs from anamorphs of most *Xylaria* species, which produce denticulate conidial secession scars that are not grouped at the top of conidiogenous cells. Anamorphs of species with a teleomorph similar to that of *X*. *furcata*, including *X*. *brevifurcata*, *X*. *scoparia*, *X*. *siamensis*, and *X*. *tenellifurcata*, can be confused with the anamorph of *X*. *furcata*. Despite having distinct anamorphic features from each other and from *X*. *furcata*, these species can still be mistaken for *X*. *furcata* especially when similar stromata from more than one *Xylaria* species appear together on a fungus comb.

### Difficulties encountered during our study of *X. furcata* and resembling species

During our study of *X*. *furcata* and resembling species, several difficulties were encountered, including: (i) a large number of the collected stromata being immature, (ii) stromata being mostly small and delicate, easily overlooked in the field, (iii) poor ascospore germination, (iv) difficulty in managing fragile stromatal tissue in small quantity during isolation, (v) small stromata drying quickly within hours and losing viability not long after, and (vi) presence of multiple similar species commonly found in a field collection. Obtaining pure cultures from multiple stromata in a collection is crucial, particularly when the presence of similar species is suspected. All of the cultures that we studied were initiated from fresh stromatal tissue before drying. Our numerous attempts to germinate ascospores on agar media were unsuccessful, as previously reported by Ju and Hsieh (2007) for other *Xylaria* species associated with termite nests in Taiwan. It is possible that the ascospore germination is triggered or significantly promoted by passage through termite guts or landing on termite fungus gardens.

## Conclusion

Macrotermitine termites are found in Africa and Asia, with a greater generic diversity in Africa. Ten species were revealed among the specimens identifiable as *X*. *furcata*, with nine known from Asia and only *X*. *hirsuta* from Africa. The diversity of *X*. *furcata* and resembling species was previously overlooked due to the small size and similarity of their stromata, resulting in *X*. *furcata* being the only recognized species. Additional undescribed species related to *X*. *furcata* have been confirmed through sequencing collections containing immature stromata (unpublished data of H-MH), indicating that the diversity is not limited to the ten species included in the present study. In fact, these ten species likely represent only a small fraction of the true diversity, with most species yet to be described. In Taiwan, *Odontotermes formosanus* is the only macrotermitine termite species; however, *X. furcata* and seven resembling species are associated with its nests. Macrotermitine termites have a known species diversity of 332, with 165 each in Africa and Asia and two from Madagascar (Kambhampati and Eggleton 2000). With the known diversity of macrotermitine termites far greater than that of *X*. *furcata* and resembling species, it is foreseeable that many more species of this fungal group are yet to be collected and described.

## Supplementary Information

**Additional file 1: Table S1.** List of isolates and taxa other than *X. furcata* and resembling species included in the phylogenetic analyses.

**Additional file 2: **Aligned ITS dataset.

**Additional file 3: **Aligned concatenated dataset of α-ACT, RPB2, and β-TUB (RPB2-TUB-ACT dataset).

## Data Availability

Specimens have been deposited at the HAST herbarium, and cultures are available at BCRC. DNA sequences have been deposited at GenBank. Newly described species and new type designations were registered at MycoBank.
