# Supplementary material for: Xylaria furcata reconsidered and nine resembling species
Source: Bot Stud. 2023 Jul 17;64:21. doi: 10.1186/s40529-023-00392-x (PMC10352222; doi:10.1186/s40529-023-00392-x)
Supplement: Supplementary file 1 — Additional file 1: Table S1. List of isolates and taxa other than X. furcata and resembling species included in the phylogenetic analyses. [file 40529_2023_392_MOESM1_ESM.docx]

Table S1. Taxa and isolates other than *X*. *furcata* and resembling species included in the phylogenetic analyses. Sequences in boldface were generated in this study.

| Taxon | Origin | Collecting data | GenBank accession number | | |  |
| --- | --- | --- | --- | --- | --- | --- |
|  |  |  | β-tubulin gene | α-actin gene | RPB2 gene | ITS |
| *Amphirosellinia fushanensis* Y.-M. Ju et al. | Taiwan | From HOLOTYPE (Ju et al. 2004) | GQ495950 | GQ452360 | GQ848339 | GU339496 |
| *Amphirosellinia nigrospora* Y.-M. Ju et al. | Taiwan | From HOLOTYPE (Ju et al. 2004) | GQ495951 | GQ452361 | GQ848340 | GU322457 |
| *Astrocystis bambusae* (Henn.) Læssøe & Spooner | Taiwan | *Ju & Hsieh 89021904* (Hsieh et al. 2010) | GQ495942 | GQ449239 | GQ844836 | GU322449 |
| *Astrocystis mirabilis* Berk. & Broome | Taiwan | *Ju & Hsieh 94070803* (Hsieh et al. 2010) | GQ495941 | GQ449238 | GQ844835 | GU322448 |
| *Astrocystis sublimbata* (Durieu & Mont.) G. C. Hughes | Taiwan | *Ju & Hsieh 89032207* (Hsieh et al. 2010) | GQ495940 | GQ449236 | GQ844834 | GU322447 |
| *Biscogniauxia arima* San Martín et al. | Mexico | *YMJ122* from ISOTYPE (Hsieh et al. 2005; Ju et al. 1998) | AY951672 | AY951784 | GQ304736 | EF026150 |
| *Discoxylaria myrmecophila* J. C. Lindq. & J. E. Wright | Mexico | *YMJ169* from *Moreno 713* (Rogers et al. 1995) | GQ487710 | GQ438747 | GQ844819 | GU322433 |
| *Entoleuca mammata* (Wahlenb.) J. D. Rogers & Y.-M. Ju | France | *YMJ100* from *Candoussau, F. 5254* (Hsieh et al. 2010) | GQ470230 | GQ398230 | GQ844782 | GU300072 |
| *Euepixylon sphaeriostomum* (Schwein.) Y.-M. Ju & J. D. Rogers | USA | *YMJ261* from *Huhndorf, S. M. 1447* (Hsieh et al. 2010) | GQ470224 | GQ389696 | GQ844774 | GU292821 |
| *Kretzschmaria clavus* (Fr.) Sacc. | French Guiana | *YMJ114* from *Huhndorf 803* (Ju et al. 2007; Rogers and Ju 1998) | EF025611 | EF025596 | GQ844789 | EF026126 |
| *Kretzschmaria guyanensis* J. D. Rogers & Y.-M. Ju | Taiwan | *Ju & Hsieh 89062903* (Hsieh et al. 2010) | GQ478214 | GQ408901 | GQ844792 | GU300079 |
| *Kretzschmaria lucidula* (Mont.) Dennis | French Guiana | *YMJ112* from *Huhndorf 677* (Ju et al. 2007; Rogers and Ju 1998) | EF025610 | EF025595 | GQ844790 | EF026125 |
| *Kretzschmaria megalospora* J. D. Rogers & Y.-M. Ju | Malaysia | *YMJ229* from *Whalley, M. FH 64-97* (Ju et al. 2007) | EF025609 | EF025594 | GQ844791 | EF026124 |
| *Kretzschmaria neocaledonica* (Har. & Pat.) J. D. Rogers & Y.-M. Ju | Taiwan | *Guu, J.-R. 94031003* (Hsieh et al. 2010) | GQ478213 | GQ398236 | GQ844788 | GU300078 |
| *Kretzschmaria pavimentosa* (Ces.) P. Martin | Taiwan | *YMJ109* from *Wang 511* (Rogers and Ju 1998) | GQ478212 | GQ398235 | GQ844787 | GU300077 |
| *Kretzschmaria sandvicensis* (Reichardt) J. D. Rogers & Y.-M. Ju | USA, Hawaiian Islands | *YMJ113* from *Rogers/4 Jan 1996* (Rogers and Ju 1998) | GQ478211 | GQ398234 | GQ844786 | GU300076 |
| *Nemania abortiva* J. D. Rogers et al. | USA, Hawaiian Islands | *YMJ467* from HOLOTYPE (Rogers et al. 2006) | GQ470219 | GQ374123 | GQ844768 | GU292816 |
| *Nemania beaumontii* (Berk. & M. A. Curtis) Y.-M. Ju & J. D. Rogers | French West Indies | *YMJ405* from *Lechat, C. CLL2039* (Fournier et al. 2018a; Hsieh et al. 2010) | GQ470222 | GQ389694 | GQ844772 | GU292819 |
| *Nemania bipapillata* (Berk. & M. A. Curtis) Pouzar | Taiwan | *Ju & Hsieh 90080610* (Hsieh et al. 2010) | GQ470221 | GQ389693 | GQ844771 | GU292818 |
| *Nemania diffusa* (Sowerby) S. F. Gray | Taiwan | *Ju & Hsieh 91020401* (Hsieh et al. 2010) | GQ470220 | GQ389692 | GQ844769 | GU292817 |
| *Nemania illita* (Schwein.) Pouzar | USA | *YMJ236* from *Tsai, S.-J.* (Ju et al. 2007) | EF025608 | EF025593 | GQ844770 | EF026122 |
| *Nemania macrocarpa* Y.-M. Ju & J. D. Rogers | USA, Hawaiian Islands | *YMJ265* from HOLOTYPE (Ju and Rogers 2002) | GQ470226 | GQ389698 | GQ844776 | GU292823 |
| *Nemania maritima* Y.-M. Ju & J. D. Rogers | Taiwan | From HOLOTYPE (Ju and Rogers 2002) | GQ470225 | GQ389697 | GQ844775 | GU292822 |
| *Nemania primolutea* Y.-M. Ju et al. | Taiwan | From HOLOTYPE (Ju et al. 2005; Ju et al. 2007) | EF025607 | EF025592 | GQ844767 | EF026121 |
| *Nemania serpens* (Pers.) S. F. Gray *“*Barron isolate” | Canada | *YMJ235* from *Barron, G.*, as *Hypoxylon* in Petrini and Rogers (1986) | GQ470223 | GQ389695 | GQ844773 | GU292820 |
| *Rosellinia buxi* Fabre | France | *YMJ99* from *Candoussau, F.* (Hsieh et al. 2010) | GQ470228 | GQ398228 | GQ844780 | GU300070 |
| *Rosellinia lamprostoma* Syd. & P. Syd. | Taiwan | *Ju & Hsieh 89112602* (Ju et al. 2007) | EF025604 | EF025589 | GQ844778 | EF026118 |
| *Rosellinia merrillii* Syd. & P. Syd. | Taiwan | *Ju & Hsieh 89112601* (Hsieh et al. 2010) | GQ470229 | GQ398229 | GQ844781 | GU300071 |
| *Rosellinia necatrix* (R. Hartig) Berl. | Taiwan | *Ju & Hsieh 89062904* (Ju et al. 2007) | EF025603 | EF025588 | GQ844779 | EF026117 |
| *Rosellinia sanctaecruciana* Ferd. & Winge | Taiwan | *Ju & Hsieh 90072903* (Hsieh et al. 2010) | GQ470227 | GQ389699 | GQ844777 | GU292824 |
| *Stilbohypoxylon elaeidicola* (Henn.) L. E. Petrini | French Guiana | *YMJ173* from *Huhndorf 928* (Ju et al. 2007), as *S. moelleri* in Rogers and Ju (1997) | EF025616 | EF025601 | GQ844826 | EF026148 |
| *Stilbohypoxylon elaeidicola* (Henn.) L. E. Petrini | Taiwan | *Ju & Hsieh 94082615* (Hsieh et al. 2010) | GQ495933 | GQ438754 | GQ844827 | GU322440 |
| *Stilbohypoxylon quisquiliarum* (Mont.) J. D. Rogers & Y.-M. Ju | French Guiana | *YMJ172* from *Huhndorf 940* (Ju et al. 2007; Rogers and Ju 1997) | EF025605 | EF025590 | GQ853020 | EF026119 |
| *Stilbohypoxylon quisquiliarum* (Mont.) J. D. Rogers & Y.-M. Ju | Taiwan | *Ju & Hsieh 89091608* (Ju et al. 2007) | EF025606 | EF025591 | GQ853021 | EF026120 |
| *Xylaria acuminatilongissima* Y.-M. Ju & H.-M. Hsieh | Taiwan | *YMJ623* from HOLOTYPE (Ju and Hsieh 2007) | GQ502711 | GQ853046 | GQ853028 | EU178738 |
| *Xylaria adscendens* (Fr.) Fr. | French West Indies | *YMJ570* from *Lechat, C. CLL5347* (Hsieh et al. 2010) | GQ487708 | GQ438745 | GQ844817 | GU300101 |
| *Xylaria adscendens* (Fr.) Fr. | Thailand | *YMJ865* from *Bandoni, R. J., Bandoni, A. A. & Flegel, T. W. 12017* (Hsieh et al. 2010) | GQ487709 | GQ438746 | GQ844818 | GU322432 |
| *Xylaria alboareolata Y.-M. Ju & J.D. Rogers* | French West Indies | *YMJ543* from *Chabrol, J. CLL5372 (Fournier et al. 2018b), as X. areolata in Hsieh et al. (2010)* | GQ478215 | GQ408902 | GQ844793 | GU300080 |
| *Xylaria allantoidea* (Berk.) Fr. | Taiwan | *Ju & Hsieh 94042903* (Hsieh et al. 2010) | GQ502692 | GQ452377 | GQ848356 | GU324743 |
| *Xylaria amphithele* San Martín & J. D. Rogers | French West Indies | *YMJ529* from *Lechat, C. CLL5352* (Hsieh et al. 2010) | GQ478218 | GQ408905 | GQ844796 | GU300083 |
| *Xylaria apoda* (Berk. & Broome) J. D. Rogers & Y.-M. Ju | Taiwan | *Ju & Hsieh 90080804* (Hsieh et al. 2010) | GQ495930 | GQ438751 | GQ844823 | GU322437 |
| *Xylaria arbuscula* Sacc. | Taiwan | *Ju & Hsieh 89041211* (Hsieh et al. 2010) | GQ478226 | GQ421286 | GQ844805 | GU300090 |
| *Xylaria arbuscula* var. *plenofissura* Y.-M. Ju & S.-S. Tzean | Taiwan | *Ju & Hsieh 93082814* (Hsieh et al. 2010) | GQ478225 | GQ421285 | GQ844804 | GU339495 |
| *Xylaria aristata* Mont. | Taiwan | *Ju & Hsieh 90071613*, as *X. sicula* f. *major* in Hsieh et al. (2010) | GQ478216 | GQ408903 | GQ844794 | GU300081 |
| *Xylaria atrodivaricata* Y.-M. Ju & H.-M. Hsieh | Taiwan | *YMJ615* from HOLOTYPE (Ju and Hsieh 2007) | GQ502713 | GQ853048 | GQ853030 | EU178739 |
| *Xylaria atrosphaerica* (Cooke & Massee) Callan & J. D. Rogers | Taiwan | *Ju & Hsieh 91111214* (Hsieh et al. 2010) | GQ495953 | GQ452363 | GQ848342 | GU322459 |
| *Xylaria badia* Pat. | Taiwan | *Ju & Hsieh 95070101* (Hsieh et al. 2010) | GQ495939 | GQ449235 | GQ844833 | GU322446 |
| *Xylaria bambusicola* Y.-M. Ju & J. D. Rogers | Taiwan | *YMJ205* from HOLOTYPE (Hsieh et al. 2005; Ju and Rogers 1999) | AY951762 | AY951873 | GQ844802 | EF026123 |
| *Xylaria bambusicola* Y.-M. Ju & J. D. Rogers | Thailand | *YMJ162* from *Bandoni, R. J. & A. A. et al.* (Hsieh et al. 2010) | GQ478223 | GQ408910 | GQ844801 | GU300088 |
| *Xylaria berteri* (Mont.) Cooke | USA, Hawaiian Islands | *YMJ256* from *Rogers, J. D. K-1* (Hsieh et al. 2010) | GQ502698 | GQ455442 | GQ848363 | GU324750 |
| *Xylaria berteri* (Mont.) Cooke | Taiwan | *Ju & Hsieh 90112623* (Hsieh et al. 2005) | AY951763 | AY951874 | GQ848362 | GU324749 |
| *Xylaria brunneovinosa* Y.-M. Ju & H.-M. Hsieh | Taiwan | *YMJ720* from HOLOTYPE (Ju and Hsieh 2007) | GQ502706 | GQ853041 | GQ853023 | EU179862 |
| *Xylaria cantareirensis* (Henn.) J. Fourn. & Lechat | French West Indies | *YMJ526* from *Lechat, C. CLL5437* (Fournier et al. 2018b), as *Penzigia* in Hsieh et al. (2010) | GQ478220 | GQ408907 | GQ844798 | GU300085 |
| *Xylaria castorea* Berk. | New Zealand | *YMJ600* from *Samuels 85-75* (Hsieh et al. 2010) | GQ502703 | GQ455447 | GQ853018 | GU324751 |
| *Xylaria* cf. *castorea* Berk. | Taiwan | *Ju & Hsieh 91092303* (Hsieh et al. 2010) | GQ502704 | GQ455448 | GQ853019 | GU324752 |
| *Xylaria chaiyaphumensis* Wangsawat et al. | Thailand | *SWUF16-11.4* (Wangsawat et al. 2021) | **OQ845433** | **OQ845425** | **OQ851585** | MT622776 |
| *Xylaria chaiyaphumensis* Wangsawat et al. | Thailand | *SWUF17-49.2* from HOLOTYPE (Wangsawat et al. 2021) | **OQ845434** | **OQ845426** | **OQ851586** | MT622775 |
| *Xylaria cirrata* Pat. | Taiwan | *YMJ664* from EPITYPE (Ju and Hsieh 2007) | GQ502707 | GQ853042 | GQ853024 | EU179863 |
| *Xylaria coccophora* Mont. | French Guiana | *YMJ786* from *Lechat, C. CLL7056* (Hsieh et al. 2010) | GQ487701 | GQ421289 | GQ844809 | GU300093 |
| *Xylaria coprinicola* Y.-M. Ju et al. | China | *YMJ1145* from HOLOTYPE (Ju et al. 2011) | HM585018 | HM585017 | HM585019 | HM585020 |
| *Xylaria cranioides* (Sacc. & Paol.) Dennis | Taiwan | *YMJ226* from *Wen 712* (Ju and Rogers 2001) | GQ478210 | GQ398233 | GQ844785 | GU300075 |
| *Xylaria crozonensis* P. Leroy & Mornand | France | *YMJ398* from *Mornand, F. JF04151* (Hsieh et al. 2010) | GQ502697 | GQ455441 | GQ848361 | GU324748 |
| *Xylaria cubensis* (Mont.) Fr. | French West Indies | *YMJ419* from *Lechat, C. CLL2179* (Fournier et al. 2019), as *X*. *laevis* in Hsieh et al. (2010) | GQ502695 | GQ455439 | GQ848359 | GU324746 |
| *Xylaria cubensis* (Mont.) Fr. | Taiwan | *Ju & Hsieh 95072910*, as *X*. *laevis* in Hsieh et al. (2010) | GQ502696 | GQ455440 | GQ848360 | GU324747 |
| *Xylaria culleniae* Berk. & Broome | Thailand | *YMJ189* from *Whalley, M. F.NH9* (Hsieh et al. 2010) | GQ495935 | GQ438756 | GQ844829 | GU322442 |
| *Xylaria cuneata* C. G. Lloyd | French West Indies | *YMJ495* from *Lechat, C. CLL5131* (Fournier et al. 2019)*,* as *X*. *montagnei* Hamme & Guerrero in Hsieh et al. (2010) | GQ495948 | GQ449244 | GQ848337 | GU322455 |
| *Xylaria curta* Fr. | French West Indies | *YMJ494* from *Lechat, C. CLL5044* (Hsieh et al. 2010) | GQ495937 | GQ449233 | GQ844831 | GU322444 |
| *Xylaria curta* Fr. | Taiwan | *Ju & Hsieh 92092022* (Hsieh et al. 2010) | GQ495936 | GQ438757 | GQ844830 | GU322443 |
| *Xylaria digitata* (L.) Grev. | Ukraine | *YMJ919* from *Prilutsky, O.* (Hsieh et al. 2010) | GQ495949 | GQ449245 | GQ848338 | GU322456 |
| *Xylaria enterogena* (Mont.) Fr. | French Guiana | *YMJ785* from *Lechat, C. CLL7043* (Hsieh et al. 2010) | GQ502685 | GQ452370 | GQ848349 | GU324736 |
| *Xylaria escharoidea* (Berk.) Fr. | Taiwan | *YMJ658* from EPITYPE (Ju and Hsieh 2007) | GQ502709 | GQ853044 | GQ853026 | EU179864 |
| *Xylaria feejeensis* (Berk.) Fr. | French West Indies | *YMJ565* from *Lechat, C. CLL5653* (Hsieh et al. 2010) | GQ495945 | GQ449241 | GQ848334 | GU322452 |
| *Xylaria feejeensis* (Berk.) Fr. | Taiwan | *Ju & Hsieh 92092013* (Hsieh et al. 2010) | GQ495947 | GQ449243 | GQ848336 | GU322454 |
| *Xylaria feejeensis* (Berk.) Fr. | Thailand | *YMJ180* from *Whalley, M. UN515* (Hsieh et al. 2010) | GQ495946 | GQ449242 | GQ848335 | GU322453 |
| *Xylaria fimbriata* C. G. Lloyd | French West Indies | *YMJ491* from *Lechat, C. CLL5010* (Hsieh et al. 2010) | GQ502705 | GQ853040 | GQ853022 | GU324753 |
| *Xylaria fissilis* Ces. | French West Indies | *YMJ367* from *Lechat, C. CLL0928* (Hsieh et al. 2010) | GQ470231 | GQ398231 | GQ844783 | GU300073 |
| *Xylaria flabelliformis* (Schwein.) Fr. | USA | *YMJ860* from *Rogers, J. D.*, as *X*. *cubensis* in Hsieh et al. (2010) | GQ502700 | GQ455444 | GQ848365 | GU991523 |
| *Xylaria flabelliformis* (Schwein.) Fr. | Papua New Guinea | *YMJ159* from *Van der Gucht & De Meester 92-521*, as *X*. *cubensis* in Van der Gucht (1995) and Hsieh et al. (2010) | GQ502702 | GQ455446 | GQ853017 | MZ854247 |
| *Xylaria flabelliformis* (Schwein.) Fr. | Russian Far East | *YMJ477* from *Vasilyeva, L. N.*, as *X*. *cubensis* in Hsieh et al. (2010) | GQ502699 | GQ455443 | GQ848364 | MZ854248 |
| *Xylaria flabelliformis* (Schwein.) Fr. | French West Indies | *YMJ515* from *Lechat, C. CLL5121* (Fournier et al. 2019), as *X*. *cubensis* in Hsieh et al. (2010) | GQ502701 | GQ455445 | GQ848366 | GU373810 |
| *Xylaria frustulosa* (Berk. & M. A. Curtis) Cooke | French West Indies | *YMJ771* from *Lechat, C. CLL6002-2* (Fournier et al. 2018b; Hsieh et al. 2010) | GQ495943 | GQ449237 | GQ844837 | GU322450 |
| *Xylaria frustulosa* (Berk. & M. A. Curtis) Cooke | Taiwan | *Ju & Hsieh 92092010* (Hsieh et al. 2010) | GQ495944 | GQ449240 | GQ844838 | GU322451 |
| *Xylaria globosa* (Spreng. ex Fr.) Mont. | French West Indies | *YMJ775* from *Lechat, C. CLL6033* (Hsieh et al. 2010) | GQ502684 | GQ452369 | GQ848348 | GU324735 |
| *Xylaria grammica* (Mont.) Fr. | Taiwan | *YMJ479* from *Chen, G.-T.* (Hsieh et al. 2010) | GQ487704 | GQ427197 | GQ844813 | GU300097 |
| *Xylaria griseosepiacea* Y.-M. Ju & H.-M. Hsieh | Taiwan | *YMJ641* from HOLOTYPE (Ju and Hsieh 2007) | GQ502714 | GQ853049 | GQ853031 | EU179865 |
| *Xylaria guepini* (Fr.) Fr. | France | *D. Huart, HD 20120101* (Hsieh et al. 2022) | OP856892 | OP856890 | OP856891 | OP863307 |
| *Xylaria haemorrhoidalis* Berk. & Broome | Taiwan | *Ju & Hsieh 89041207* (Hsieh et al. 2010) | GQ502683 | GQ452368 | GQ848347 | GU322464 |
| *Xylaria* cf. *heliscus* (Mont.) J. D. Rogers & Y.-M. Ju | Taiwan | *Ju & Hsieh 88113010* (Hsieh et al. 2010) | GQ502691 | GQ452376 | GQ848355 | GU324742 |
| *Xylaria hypoxylon* (L.) Grev. | Belgium | *YMJ152* from *Ju, Y.-M.* (Hsieh et al. 2010) | GQ260187 | GQ427196 | GQ844812 | GU300096 |
| *Xylaria hypoxylon* (L.) Grev. | Taiwan | *Guu, J.-R. 95082001* (Hsieh et al. 2010) | GQ487703 | GQ427195 | GQ844811 | GU300095 |
| *Xylaria ianthinovelutina* (Mont.) Fr. | French West Indies | *YMJ553* from *Lechat, C. CLL5599* (Hsieh et al. 2010) | GQ495934 | GQ438755 | GQ844828 | GU322441 |
| *Xylaria insolita* Y.-M. Ju et al. | Taiwan | *YMJ1251* from HOLOTYPE (Hsieh et al. 2020) | MN656983 | MN656985 | MN656981 | MN655979 |
| *Xylaria intracolorata* (J. D. Rogers et al.) J. D. Rogers & Y.-M. Ju | Taiwan | *Ju & Hsieh 90080402* (Hsieh et al. 2010) | GQ502690 | GQ452375 | GQ848354 | GU324741 |
| *Xylaria intraflava* Y.-M. Ju & H.-M. Hsieh | Taiwan | *YMJ725* from HOLOTYPE (Ju and Hsieh 2007) | GQ502718 | GQ853053 | GQ853035 | EU179866 |
| *Xylaria ischnostroma* Wangsawat et al. | Thailand | *SWUF18-22.1* from HOLOTYPE (Wangsawat et al. 2021) | **OQ845435** | **OQ845427** | **OQ851587** | MT622788 |
| *Xylaria juruensis* Henn. | Taiwan | *Ju & Hsieh 92042501* (Hsieh et al. 2010) | GQ495932 | GQ438753 | GQ844825 | GU322439 |
| *Xylaria liquidambar* J. D. Rogers et al. | Taiwan | *Ju & Hsieh 93090701* (Hsieh et al. 2010) | GQ487702 | GQ421290 | GQ844810 | GU300094 |
| *Xylaria luteostromata* C. G. Lloyd var*. macrospora* J. D. Rogers & Samuels | French West Indies | *YMJ508* from *Lechat, C. CLL5020* (Hsieh et al. 2010) | GQ502688 | GQ452373 | GQ848352 | GU324739 |
| *Xylaria meliacearum* Læssøe | Puerto Rico | *YMJ148* from *Lodge, D. J. PR-894* (Læssøe and Lodge 1994) | GQ478219 | GQ408906 | GQ844797 | GU300084 |
| *Xylaria mianyangensis* Y.-M. Ju et al. | China | *YMJ1321* from HOLOTYPE (the present study) | MZ901319 | MZ901347 | MZ901333 | MZ888986 |
| *Xylaria microceras* (Mont.) Fr. | French West Indies | *YMJ414* from *Lechat, C. CLL2265* (Hsieh et al. 2010) | GQ478221 | GQ408908 | GQ844799 | GU300086 |
| *Xylaria multiplex* (Kunze) Fr. | French West Indies | *YMJ580* from *Lechat, C. CLL5287* (Hsieh et al. 2010) | GQ487705 | GQ427198 | GQ844814 | GU300098 |
| *Xylaria minima* Wangsawat et al. | Thailand | *SWUF18-3.2* from HOLOTYPE (Wangsawat et al. 2021) | **OQ845436** | **OQ845428** | **OQ851588** | MT622789 |
| *Xylaria multiplex* (Kunze) Fr. | USA, Hawaiian Islands | *YMJ259* from *Hemmes, D. E. Xy-7* (Hsieh et al. 2010) | GQ487706 | GQ438743 | GQ844815 | GU300099 |
| *Xylaria muscula* C. G. Lloyd | French West Indies | *YMJ520* from *Lurel, D. CLL5323* (Hsieh et al. 2010) | GQ478222 | GQ408909 | GQ844800 | GU300087 |
| *Xylaria neonigripes* Y.-M. Ju et al. | China | *WLS2030* (the present study) | MZ901320 | MZ901348 | MZ901334 | MZ888987 |
| *Xylaria neonigripes* Y.-M. Ju et al. | Taiwan | *YMJ722* from *Chou, K.-H. 95060503,* as *X*. sp. 3 in Hsieh et al. (2010) | GQ502712 | GQ853047 | GQ853029 | GU324756 |
| *Xylaria neonigripes* Y.-M. Ju et al. | Taiwan | *YMJ1202* from HOLOTYPE (the present study) | MZ901321 | MZ901349 | MZ901335 | MZ888988 |
| *Xylaria nigripes* (Klotzsch) Fr. | China | *WLS2056* (the present study) | MZ901322 | MZ901350 | MZ901336 | MZ888989 |
| *Xylaria nigripes* (Klotzsch) Fr. | Taiwan | *YMJ653* from *Chou, K.-H. 94053001* (Ju and Hsieh 2007) | GQ502710 | GQ853045 | GQ853027 | EU179868 |
| *Xylaria ochraceostroma* Y.-M. Ju & H.-M. Hsieh | Taiwan | *YMJ401* from HOLOTYPE (Ju and Hsieh 2007) | GQ502717 | GQ853052 | GQ853034 | EU179869 |
| *Xylaria oligotoma* Sacc. & Paol. | French Guiana | *YMJ784* from *Lechat, C. CLL7031* (Hsieh et al. 2010) | GQ487700 | GQ421288 | GQ844808 | GU300092 |
| *Xylaria ophiopoda* Sacc. | Taiwan | *Ju & Hsieh 93082805* (Hsieh et al. 2010) | GQ495955 | GQ452365 | GQ848344 | GU322461 |
| *Xylaria oxyacanthae* Tul. & C. Tul. | USA | *YMJ859* from *Yeomans, R.* (Hsieh et al. 2010) | GQ495927 | GQ438748 | GQ844820 | GU322434 |
| *Xylaria palmicola* G. Winter | New Zealand | *YMJ604* from *Samuels, G. J. 85-83* (Hsieh et al. 2010) | GQ495929 | GQ438750 | GQ844822 | GU322436 |
| *Xylaria papulis* C. G. Lloyd | Taiwan | *Ju & Hsieh 89021903* (Hsieh et al. 2010) | GQ487707 | GQ438744 | GQ844816 | GU300100 |
| *Xylaria* *phyllocharis* Mont. | French West Indies | *YMJ528* from *Lechat, C. CLL5302* (Hsieh et al. 2010) | GQ495938 | GQ449234 | GQ844832 | GU322445 |
| *Xylaria plebeja* Ces. | Taiwan | *Ju & Hsieh 91122401* (Hsieh et al. 2010) | GQ502689 | GQ452374 | GQ848353 | GU324740 |
| *Xylaria polymorpha* (Pers.) Grev. | USA | *YMJ1012* from *Rogers, J. D.* (Hsieh et al. 2010) | GQ495954 | GQ452364 | GQ848343 | GU322460 |
| *Xylaria reevesiae* Y.-M. Ju et al. | Taiwan | From HOLOTYPE (Ju et al. 2018), as *X*. sp. 7 in Hsieh et al. (2010) | GQ495928 | GQ438749 | GQ844821 | GU322435 |
| *Xylaria regalis* Cooke | India | *YMJ920* from *Gailawad, S. AMH 9204* (Hsieh et al. 2010) | GQ502694 | GQ452379 | GQ848358 | GU324745 |
| *Xylaria regalis* Cooke | Taiwan | *Ju & Hsieh 92072001* (Hsieh et al. 2010) | GQ502693 | GQ452378 | GQ848357 | GU324744 |
| *Xylaria rhytidosperma* J. Fourn. & Lechat | French West Indies | *YMJ431* from ISOTYPE (Fournier et al. 2018b), as *X*. cf. *glebulosa* in Hsieh et al. (2010) | GQ495956 | GQ452366 | GQ848345 | GU322462 |
| *Xylaria rogersionigripes* Y.-M. Ju et al. | China | *WLS2064* (the present study) | MZ901323 | MZ901351 | MZ901337 | MZ888990 |
| *Xylaria rogersionigripes* Y.-M. Ju et al. | Taiwan | *YMJ1791* from HOLOTYPE (the present study) | MZ901324 | MZ901352 | MZ901338 | MZ888991 |
| *Xylaria rogersionigripes* Y.-M. Ju et al. | Taiwan | *YMJ1795* from HOLOTYPE (the present study) | MZ901325 | MZ901353 | MZ901339 | MZ888992 |
| *Xylaria schweinitzii* Berk. & M. A. Curtis | Taiwan | *Ju & Hsieh 92092023* (Hsieh et al. 2010) | GQ495957 | GQ452367 | GQ848346 | GU322463 |
| *Xylaria scruposa* (Fr.) Fr. | French West Indies | *YMJ497* from *Lechat, C. CLL5025* (Hsieh et al. 2010) | GQ495952 | GQ452362 | GQ848341 | GU322458 |
| *Xylaria* sp. A | China | *WLS2062* (the present study) | MZ901326 | MZ901354 | MZ901340 | MZ888993 |
| *Xylaria* sp. A | China | *WLS2069* (the present study) | MZ901327 | MZ901355 | MZ901341 | MZ888994 |
| *Xylaria* sp. A | China | *WLS2077* (the present study) | MZ901328 | MZ901356 | MZ901342 | MZ888995 |
| *Xylaria* sp. B | China | *WLS2084* (the present study) | MZ901329 | MZ901357 | MZ901343 | MZ888996 |
| *Xylaria striata* Pat. | China | *YMJ304* from *Leu, L.-S.* (Hsieh et al. 2010) | GQ478224 | GQ421284 | GQ844803 | GU300089 |
| *Xylaria subescharoidea* Y.-M. Ju et al. | China | *YMJ1442* (the present study) | MZ901330 | MZ901358 | MZ901344 | MZ888997 |
| *Xylaria subescharoidea* Y.-M. Ju et al. | China | *YMJ1484* (the present study) | MZ901331 | MZ901359 | MZ901345 | MZ888998 |
| *Xylaria subescharoidea* Y.-M. Ju et al. | China | *WLS2026* (the present study) | MZ901332 | MZ901360 | MZ901346 | MZ888999 |
| *Xylaria subescharoidea* Y.-M. Ju et al. | Taiwan | *YMJ1188* from HOLOTYPE (Hsieh et al. 2020) | MN656984 | MN656986 | MN656982 | MN655980 |
| *Xylaria subescharoidea* Y.-M. Ju et al. | Taiwan | *YMJ660* from *Chou, K.-H. 95052301*, as *X*. sp. 2 in Hsieh et al. (2010); immature | GQ502708 | GQ853043 | GQ853025 | GU324754 |
| *Xylaria subintraflava* Wangsawat et al. | Thailand | *SWUF16-4.3* (Wangsawat et al. 2021) | **OQ845438** | **OQ845430** | **OQ851590** | MT622762 |
| *Xylaria telfairii* (Berk.) Fr. | French West Indies | *YMJ421* from *Lechat, C. CLL2224* (Fournier et al. 2019; Hsieh et al. 2010) | GQ502686 | GQ452371 | GQ848350 | GU324731 |
| *Xylaria telfairii* (Berk.) Fr. | Taiwan | *Ju & Hsieh 90081901* (Hsieh et al. 2010) | GQ502687 | GQ452372 | GQ848351 | GU324738 |
| *Xylaria terricola* Y.-M. Ju et al. | Taiwan | *YMJ1375* from HOLOTYPE (Chou et al. 2017) | MF577044 | MF577045 | MF577043 | MF577042 |
| *Xylaria terricola* Y.-M. Ju et al. | Taiwan | *Chou, W.-N.* *CWN08372* (Chou et al. 2017) | MF577040 | MF577041 | MF577039, | MF577038, |
| *Xylaria theinhirunae* Wangsawat et al. | Thailand | *SWUF16-10.1* (Wangsawat et al. 2021) | **OQ845439** | **OQ845431** | **OQ851591** | MT622773 |
| *Xylaria theinhirunae* Wangsawat et al. | Thailand | *SWUF17-44.1* from HOLOTYPE (Wangsawat et al. 2021) | **OQ845440** | **OQ845432** | **OQ851592** | MT622771 |
| *Xylaria tuberoides* Rehm | French West Indies | *YMJ475* from *Lechat, C. CLL2146* (Fournier et al. 2019; Hsieh et al. 2010) | GQ478209 | GQ398232 | GQ844784 | GU300074 |
| *Xylaria vagans* Petch | USA, Hawaiian Islands | *YMJ258* from *Hemmes, D. E. DEH-1052, as X.* sp. 6 in Hsieh et al. (2010) | GQ478217 | GQ408904 | GQ844795 | GU300082 |
| *Xylaria venosula* Speg. | USA, Hawaiian Islands | *Ju & Hsieh 94080508* (Ju et al. 2007) | EF025617 | EF025602 | GQ844806 | EF026149 |
| *Xylaria venustula* Sacc. | Taiwan | *Ju & Hsieh 88113002* (Hsieh et al. 2010) | GQ487699 | GQ421287 | GQ844807 | GU300091 |
| *Xylaria* *vivantii* Y.-M. Ju et al. | French West Indies | *YMJ519* from HOLOTYPE (Ju et al. 2018), as *X*. sp. 8 in Hsieh et al. (2010) | GQ495931 | GQ438752 | GQ844824 | GU322438 |

References

Chou W-N, Hsieh H-M, Ju Y-M (2017) *Xylaria terricola* sp. nov., a terrestrial anamorphic *Xylaria* species found in Taiwan. Fungal Science 32:1–8

Fournier J, Lechat C, Courtecuisse R (2018a) The genera *Kretzschmariella* and *Nemania* (Xylariaceae) in Guadeloupe and Martinique (French West Indies). Ascomycete.org 10:1–47

--- (2018b) The genus *Xylaria* sensu lato (Xylariaceae) in Guadeloupe and Martinique (French West Indies) I. Taxa with penzigioid stromata. Ascomycete.org 10:131–176

--- (2019) The genus *Xylaria* sensu lato (Xylariaceae) in Guadeloupe and Martinique (French West Indies) II. Taxa with robust upright stromata. Ascomycete.org 11:77–115

Hsieh H-M, Ju Y-M, Rogers JD (2005) Molecular phylogeny of *Hypoxylon* and closely related genera. Mycologia 97:844–865

Hsieh H-M, Lin C-R, Fang M-J, Rogers JD, Fournier J, Lechat C, Ju Y-M (2010) Phylogenetic status of *Xylaria* subgen. *Pseudoxylaria* among taxa of the subfamily Xylarioideae (Xylariaceae) and phylogeny of the taxa involved in the subfamily. Molecular Phylogenetics and Evolution 54:957–969

Hsieh H-M, Chou J-C, Ju Y-M (2020) *Xylaria insolita* and *X. subescharoidea*: two newly described species collected from a termite nesting site in Hua-lien, Taiwan. Botanical Studies 61:11:1–9

Hsieh H-M, Ju Y-M, Lechat C, Fournier J, Huart D (2022) New ecological, morphological, cultural and molecular phylogenetic insights into *Xylaria guepini* (Xylariaceae). Ascomycete.org 14:177–184

Ju Y-M, Rogers JD, San Martin F, Granmo A (1998) The genus *Biscogniauxia*. Mycotaxon 66:1–98

Ju Y-M, Rogers JD (1999) The Xylariaceae of Taiwan (excluding *Anthostomella*). Mycotaxon 73:343–440

--- (2001) *Xylaria cranioides* and *Poronia pileiformis* and their anamorphs in culture, and implications for the status of *Penzigia*. Mycological research 105:1134–1136

--- (2002) The genus *Nemania*. Nova Hedwigia 74:75–120

Ju Y-M, Rogers JD, Hsieh H-M (2004) *Amphirosellinia* gen. nov. and a new species of *Entoleuca*. Mycologia 96:1393–1402

--- (2005) New *Hypoxylon* and *Nemania* species from Costa Rica and Taiwan. Mycologia 97:562–567

Ju Y-M, Hsieh H-M (2007) *Xylaria* species associated with nests of *Odontotermes formosanus* in Taiwan. Mycologia 99:936–957

Ju Y-M, Hsieh H-M, Ho M-C, Szu D-H, Fang M-J (2007) *Theissenia rogersii* sp. nov. and phylogenetic position of *Theissenia*. Mycologia 99:612–621

Ju Y-M, Hsieh H-M, He X-S (2011) *Xylaria coprinicola*, a new species that antagonizes cultivation of *Coprinus comatus* in China. Mycologia 103:424–430

Ju Y-M, Rogers JD, Hsieh H-M (2018) *Xylaria* species associated with fallen fruits and seeds. Mycologia 110:726–749

Læssøe T, Lodge DJ (1994) Three host specific *Xylaria* species. Mycologia 86:436–446

Petrini L, Rogers JD (1986) A summary of the *Hypoxylon serpens* complex. Mycotaxon 26:401–436

Rogers JD, Ju Y-M, San Martin F (1995) *Discoxylaria myrmecophila* and its *Hypocreodendron* anamorph. Mycologia 87:41–45

Rogers JD, Ju Y-M (1997) The genus *Stilbohypoxylon*. Mycological research 101:135–138

--- (1998) The genus *Kretzschmaria*. Mycotaxon 68:345–393

Rogers JD, Ju Y-M, Hemmes DE (2006) *Hypoxylon subdisciforme* sp. nov., *Nemania abortiva* sp. nov., and *Xylotumulus gibbisporus* gen. et sp. nov. from Hawaii, Hawaiian Islands. Sydowia 58:290–299

Van der Gucht K (1995) Illustrations and descriptions of xylariaceous fungi collected in Papua New Guinea. Bulletin du Jardin Botanique National de Belgique 64:219–403

Wangsawat N, Ju Y-M, Phosri C, Whalley AJS, Suwannasai N (2021) Twelve new taxa of *Xylaria* associated with termite nests and soil from northeast Thailand. Biology-Basel 10:575:1–33
